# Four New C9 Metabolites from the Sponge-Associated Fungus *Gliomastix* sp. ZSDS1-F7-2

**DOI:** 10.3390/md16070231

**Published:** 2018-07-09

**Authors:** Jun Zhang, Zhiqiang Yang, Yan Liang, Liping Zhong, Huiting Lin, Balian Zhong, Liangchun Li, Shihai Xu, Yonghong Liu

**Affiliations:** 1 National Engineering Research Center of Navel Orange, Gannan Normal University, Ganzhou 341000, China; 15707975909@163.com (Z.Y.); zjzzf2011@gmail.com (Y.L.); 18270045477@163.com (L.Z.); lht18250202553@163.com (H.L.); bal.zh@163.com (B.Z.); 2 School of Life Science and Engineering, Southwest University of Science and Technology, Mianyang 621010, China; 3 Department of Chemistry, Jinan University, Guangzhou 510000, China; 4 Center for Marine Microbiology, South China Sea Institute of Oceanology, Chinese Academy of Sciences, Guangzhou 510000, China; yonghongliu@scsio.ac.cn

**Keywords:** *Gliomastix* sp., sponge-associated fungus, γ-lactone, δ-lactone, C9 metabolites

## Abstract

Four new structurally related metabolites, one γ-lactone named gliomasolide F (**1**), one δ-lactone named gliomasolide G (**2**), and two medium-chain fatty acids named gliomacids A–B (**3**–**4**), each containing nine carbons in total, were identified from the sponge-associated fungus *Gliomastix* sp. ZSDS1-F7-2. The planar chemical structures of these novel C9 metabolites were elucidated by nuclear magnetic resonance (NMR) spectroscopic methods, in connection with the analysis of high-resolution mass spectrometry (HRMS) and infrared (IR) data. The absolute configuration of **1**, was determined by comparisons of experimental circular dichroism (CD) and optical rotation (OR) value with corresponding ones computed by quantum chemistry. The relative configuration of **2** was determined by the Nuclear Overhauser effect spectroscopy (NOESY) spectrum, while its absolute configuration was tentatively determined in view of the biogenetic and biosynthetic relationships between **1** and **2**. Compounds **3**–**4**, originally as an inseparable mixture, were successfully isolated after chemical modifications. The stereo-chemistries of compounds **3**–**4** were assumed by comparison of ^13^C NMR with those of the similar moiety reported in literature, in addition to the biogenetic and biosynthetic relationships with **1**. The plausible biosynthetic relationships among these four C9 metabolites were supposed. Biologically, compounds **1**–**4** showed no cytotoxic effect against HeLa cell line at concentrations up to 25 μg/mL, while **1** exhibited moderate antifouling activity against the settlement of *Balanus amphitrite* larvae with IC_50_ being 12.8 μg/mL and LC_50_ > 25 μg/mL. The co-occurrence of macrolides gliomasolides A—E and four C9 metabolites in the same fermentation culture made us assume that these C9 metabolites might be biosynthetic building blocks toward the construction of more complex macrolides such as gliomasolides A—E or other unidentified polyketides.

## 1. Introduction

Marine sponge-associated microorganisms, known as one of the most prolific resources for the discovery of chemical and biological diversified secondary metabolites, have been and continue to be among the research interests of chemists and biologists worldwide [1,2,3,4,5,6,7]. During the past decades, a myriad of structurally unique and pharmacologically interesting drug leads have been identified from the microorganisms derived from varieties of marine sponges, involving diterpenes inhibiting the growth of a panel of cancer cell lines from a fungus *Trichoderma harzianum* OUPS-111D-4 separated from a marine sponge *Halichondria okadai* [8], antibactetial and antifungal isocoumarins from a culture of the fungus *Pestalotiopsis heterocornis* associated with sponge *Phakellia fusca* [9], cytotoxic lactones from the sponge-derived fungus *Talaromyces rugulosus* [10], and cyclopentenones with *β*-amyloid fibrillization inhibitory effects from a sponge-derived fungus *Trichoderma* sp. HPQJ-34 [11].

**Figure 1:**
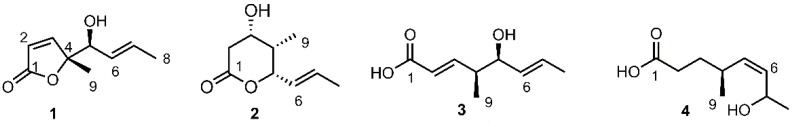
Structures of compounds **1**–**4**.

As part of our continuous effort to identify structurally and biologically interesting natural products from marine resources [12,13,14], the fungus *Gliomastix* sp. ZSDS1-F7-2 from the marine sponge *Phakellia fusca* Thiele, which was collected from the South China Sea in March 2012, has been investigated with the discovery of a series of unusual macrolides gliomasolides A—E with cytotoxic effect against the growth of HeLa cell line [15]. To further dig out bioactive metabolites from the remaining fractions of the same crude extract, we carried out systematically purification work, resulting in the discovery of four new metabolites (**1**–**4**), each having nine carbons in total, briefly named as C9 metabolites (Figure 1).

**Scheme 1:**
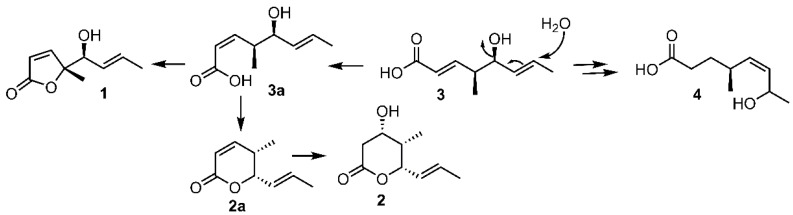
Assumed biosynthetic relationships of **1**–**4**.

Interestingly, these four C9 metabolites represented three kinds of structure-related skeletons. Compound **1** was an unusual new γ-lactone which differed from other γ-lactones mainly by the presence of a methyl group in the γ position of the ester carbonyl. **2** represented a new δ-lactone, while compounds **3**–**4** were two new medium-chain fatty acids. In view of their structure similarity, a plausible biosynthetic relationship was assumed. As depicted in Scheme 1, compound **3** might shift the double bond and hydroxyl group by allylic alcohol rearrangement, followed by hydrogenation of the conjugated double bond to give **4**. The intramolecular lactonisation of **3a** which was formed by *trans-cis* isomerization of **3**, at the γ position furnished 1, while cyclization of 3a at the δ position giving the δ-lactone **2a**, which might undergo Michael addition by water to give **2**. The co-occurrence of macrolides gliomasolides A—E and four C9 metabolites in the same fermentation culture made us assume that these C9 metabolites might be biosynthetic building blocks toward the construction of more complex macrolides such as gliomasolides A—E or other unidentified polyketides. This assumption was consistent with the widely accepted hypothesis of step by step functionalization of growing polyketide chains [16,17]. Biologically, these novel C9 metabolites exhibited no cytotoxic effect against HeLa cell line at concentrations up to 25 μg/mL, while **1** exhibited moderate antifouling activity against the settlement of *Balanus amphitrite* larvae with IC_50_ being 12.8 μg/mL and LC_50_ > 25 μg/mL. This paper reported the isolation, structural elucidation, biosynthetic relationship, and biological effects of four new C9 metabolites from the fungus *Gliomastix* sp. ZSDS1-F7-2.

## 2. Results and Discussion

**Figure 2:**
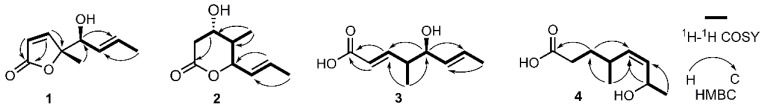
Key ^1^H–^1^H correlation spectroscopy (COSY) and heteronuclear multiple-bond correlation spectroscopy (HMBC) correlations of **1**–**4**.

Compound **1** was obtained as a brown oil. The molecular formula of **1** was determined to be C_9_H_12_O_3_ by high-resolution time-of-flight mass spectrometry (HR-TOF-MS), displaying ion peak [M + H]^+^ at *m*/*z* 169.0872 (calcd. for C_9_H_13_O_3_^+^ at *m*/*z* 169.0865). The infrared (IR) spectrum of compound **1** showed absorption bands for hydroxyl group (3487 cm^−1^) and carbonyl group (1746 cm^−1^). Preliminary inspection of the ^1^H nuclear magnetic resonance (NMR) spectrum of **1** led to the clear identification of a singlet methyl group at δ_H_ 1.40 (3H, s, H-9), a doublet methyl at δ_H_ 1.70 (3H, d, *J* = 6.0 Hz, H-8) in the upfield area (Table 1). The presence of an oxygenated methine in **1**, was clearly suggested by the chemical shifts of δ_H_ 4.14 (1H, d, *J* = 6.9 Hz, H-5) and the corresponding δ_C_ 75.8 in the ^1^H and ^13^C NMR, respectively. In the downfield region of the ^1^H NMR, the signals at δ_H_ 6.05 (1H, d, *J* = 5.4 Hz, H-2), 7.40 (1H, d, *J* = 5.4 Hz, H-3), 5.43 (1H, dd, *J* = 15.0, 6.9 Hz, H-6) and 5.78 (1H, dq, *J* = 15.0, 6.0 Hz, H-7), combined with the corresponding ^13^C NMR signals at δ_C_ 121.8 (C-2), 158.5 (C-3), 127.6 (C-6), 131.0 (C-7), supported the presence of two pairs of olefinic groups in **1**. The linear fragment extending from C-5 to C-8, was established by the ^1^H-^1^H correlation spectroscopy (COSY) showing sequential correlations of between H-5 with H-6, H-6 with H-7, and H-7 with H-8, which was further supported by the correlations of from H-5 to C-7, H-8 to C-6/C-7 in heteronuclear multiple-bond correlation spectroscopy (HMBC) (Figure 2). The fragment extending from C-1 to C-4 in **1**, was established by the HMBC correlations of from both H-2/H-3 to C-1/C-4, as shown in Figure 2. Whereas, the connection between C-1 with C-4 via an oxygen atom to form the butenolide moiety was deduced by the high-resolution mass spectrometry (HRMS) spectrum referring the molecular formula of C_9_H_12_O_3_ to **1**, requiring the cyclization to satisfy the unsaturated degrees. This assignment was also in agreement with the IR absorbance of carbonyl carbon at 1746 cm^−1^ which was found at a much higher wavenumber than the α,β unsaturated acid (around 1700 cm^−1^). The arrangement of a singlet methyl group connected to the C-4, was determined by the correlations of from H-9 to C-4/C-3/C-5 in HMBC (Figure 2). Furthermore, the connection of the moiety extending from C-5 to C-8, and the butenolide moiety extending from C-1 to C-4, to form the complete planar structure of **1**, as shown in Figure 1, was established by the HMBC correlations between H-5 and C-3/C-4/C-9, and between H-3 and C-4/C-5/C-9, respectively.

**Figure 3:**
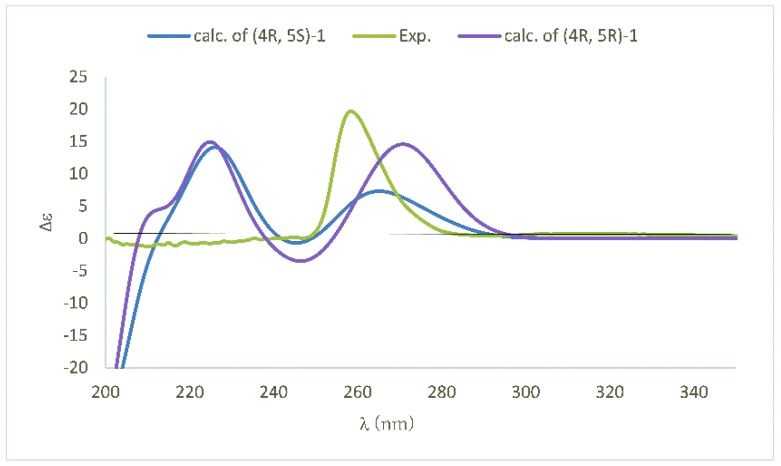
Experimental and calculated circular dichroism (CD) spectra of **1**.

The stereochemistry of the double bond Δ^6,7^ was determined to be *trans* in view of the coupling constant of *J*_6,7_ = 15.0 Hz. To determine the stereo-orientation of the hydroxyl group at C-5, the Mosher ester method was employed. According to the procedure previously reported by our group, the (*S*)-MTPA (methoxy(trifluoromethyl)phenylacetic acid) ester was successfully obtained, unfortunately, in spite of modifying the reaction conditions several times, we failed to obtain the (*R*)-MTPA ester, which made our effort using the Mosher ester method in vain. Pleasingly, the absolute configurations of both chiral carbons C-4/C-5, were successfully determined by the quantum chemical method using TDDFT (time-dependent density functional theory) calculations. Theoretically, there are four configurations for the structure of **1**, being (4*R*, 5*S*)-**1**, (4*R*, 5*R*)-**1** and their enantiomers. The absolute configuration of **1** was determined by comparisons of the calculated CD (circular dichroism) and OR (optical rotation) value with those of the corresponding experimental data. The calculated CD spectra of both (4*R*, 5*S*)-**1** and (4*R*, 5*R*)-**1** were similar to the experimental spectrum of **1** (Figure 3), excluding the possibility of assigning their enantiomers to **1**. Furthermore, the OR values of (4*R*, 5*S*)-**1** and (4*R*, 5*R*)-**1** were calculated at the B3LYP/6-311++g (2d, p) level using methanol as solvent. The computed OR values of (4*R*, 5*S*)-**1** and (4*R*, 5*R*)-**1** were +99.6 and +241.6, respectively, which supported the assignment of (4*R*, 5*S*) stereochemistry to **1** in view of the information that the computed OR value of (4*R*, 5*S*)-**1** was more close to the experimental value (+88.3) than that of (4*R*, 5*R*)-**1**. Thus, based on both the electronic circular dichroism (ECD) and OR calculations, the structure with absolute configuration of **1** was determined to be (*R*)-5-((*S*,*E*)-1-hydroxybut-2-en-1-yl)-5-methylfuran-2(5*H*)-one, trivially named gliomasolide F.

**Figure 4:**
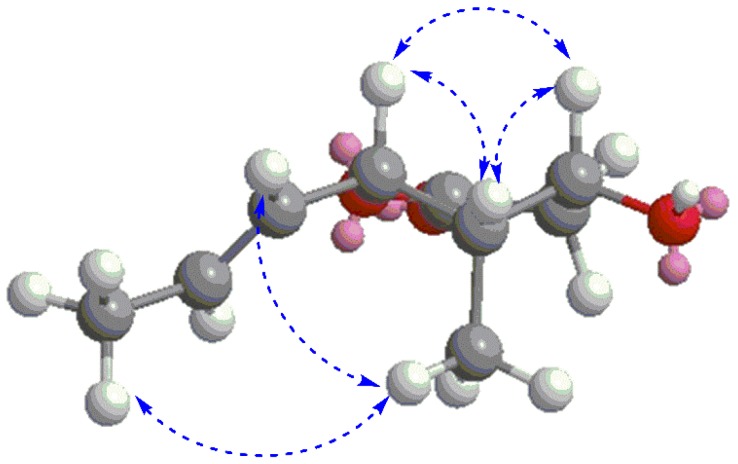
Key nuclear Overhauser effect (NOE) correlations of **2**.

Compound **2** was obtained as a brown oil. Its molecular formula C_9_H_14_O_3_ was determined by HR-TOF-MS displaying ion peaks at *m*/*z* 171.1029 [M + H]^+^ (calcd. for C_9_H_15_O_3_^+^: 171.1021) and 153.0932 [M − H_2_O + H]^+^ (calcd. for C_9_H_13_O_2_^+^: 153.0916). The IR spectrum suggested the presence of hydroxyl (3503 cm^−1^) and conjugated ester groups (1718 cm^−1^). The NMR data of **2** was similar to that of prelactone C first isolated from the *Streptomyces* sp. [17], with the major difference arising from the signals of CH-3, CH-4, CH-5, and CH_3_-9, as indicated in Table S1. The planar structure of **2** was unambiguously determined to be the same as that of prelactone C based on the comprehensive elucidation of the 2D NMR involving ^1^H–^1^H COSY, heteronuclear single quantum correlation (HSQC) and HMBC. The relative stereochemistry of chiral carbons C-3/C-4/C-5 was established according to the nuclear Overhauser effect spectroscopy (NOESY) spectrum in which the correlations of H-3 with both H-4 and H-5, H-9 with both H-6 and H-8 were clearly identified (Figure 4), allowing the assignment of all-*cis* configurations for hydroxyl at C-3, methyl at C-4, and propenyl at C-5, as shown in Figure 1. The absolute configurations of 3*S*, 4*S* and 5*S* were tentatively assigned to **2** based on the biogenetic point of view, in combination with the plausible biosynthetic relationship between **1** and **2** (Scheme 1). Worthy of note, the carbon chemical shift of CH_3_-9 in **2** resonated at δ_C_ 5.9, significantly upfield shifted compared to that of its isomer prelactone C at δ_C_ 13.7 [17]. We assumed that, due to the hydroxyl, propenyl, and methyl groups all being in *cis*-relationship, they were close to each other in spatial distance, making both the hydroxyl and propenyl groups exhibit strong gamma gauche effect which was generally observed causing an upfield shift of the resonating carbon [18,19], resulting in significant shield effect on methyl. Therefore, the chemical structure of **2** was tentatively elucidated, trivially named gliomasolide G.

**Scheme 2:**
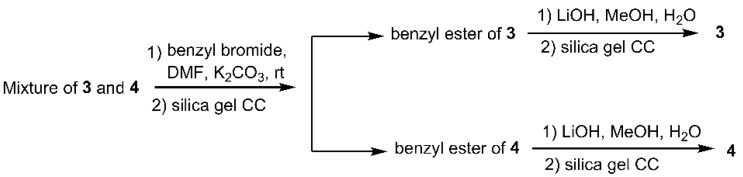
Chemical modifications of **3** and **4**.

Based on NMR analysis, compounds **3** and **4** were found to be mixed in one thin-layer chromatography (TLC) fraction, with a rough 1:1 ratio, which were then subjected to prep-high-performance liquid chromatography (HPLC) for purification. However, in spite of trying varieties of HPLC conditions, these two compounds could not be separated well. After carefully examining the NMR spectra and the HRMS data, we suspected that this mixture should consist of a pair of medium-chain fatty acids, inspiring us to esterify them before processing separation. Fortunately, after esterification using benzyl bromide, their corresponding esters were successfully separated by silica gel column chromatography, which were then subjected to LiOH solution to remove the benzyl group, giving the pure compounds **3** and **4** (Scheme 2).

**Figure 5:**
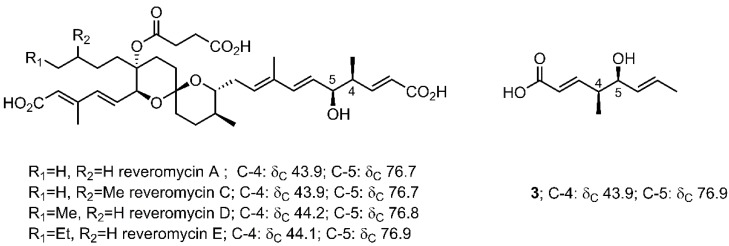
Comparison of ^13^C NMR data of reveromycins and **3**.

Compound **3** was obtained as a yellow oil, and its molecular formula C_9_H_14_O_3_ was established by the ^1^H, ^13^C NMR data, in combination with the HRMS displaying ion peaks at *m*/*z* 153.0924 [M − H_2_O + H]^+^ (calcd. for C_9_H_13_O_2_^+^: 153.0916). The IR displayed typical linear α,β-unsaturated carbonyl carbon absorption at 1697 cm^−1^, in agreement with the observation of chemical shift of δ_C_ 171.2 (C-1) in the ^13^C NMR spectrum. The ^1^H, ^13^C NMR data, indicated the presence of four olefinic protons and carbons, one oxygenated methine, and two doublet methyls (Table 2). The ^1^H–^1^H COSY correlations sequentially extending from H-2 to H-9, combined with the correlations of from H-2/H-3 with C-1, H-9 with C-3/C-5, H-5 with C-9/C-7, and H-8 with C-6/C-7 in HMBC, clearly constructed the planar structure of **3**, shown in Figure 1. The geometric configurations of both the double bonds Δ^2,3^, Δ^6,7^ were determined to be *trans* by the coupling constants, being 15.0 Hz for both *J*_2,3_ and *J*_6,7_. The relative configurations, with hydroxyl and methyl CH_3_-9 being assigned as *cis*, was established by comparison of ^13^C NMR data between **3** and the similar moiety of reveromycins, a series of polyketide spiroketals from *Streptomyces* sp. [20]. The reveromycins contained a medium-chain acid, being very similar to **3**, as its structure moiety. As shown in Figure 5, the carbon chemical shifts of C-4 and C-5 in all reveromycins resonated at ~44.0 and ~76.9 ppm in CD_3_OD, respectively, almost the same as those corresponding signals in **3** with C-4 and C-5 at δ_C_ 43.9 and 76.9, respectively. The absolute configurations 4*S*/5*S*, were tentatively assigned in view of the biogenetic and biosynthetic relationships between **1** and **3**. Therefore, the chemical structure of compound **3** was achieved, being (2*E*,4*S*,5*S*,6*E*)-5-hydroxy-4-methylocta-2,6-dienoic acid, trivially named gliomacid A.

Compound **4**, structurally similar to **3**, has nine carbons in total, involving one carbonyl, two olefinic carbons and one oxygenated methine, as revealed by ^13^C NMR. The HRMS displayed ion peaks at *m*/*z* 155.1073 [M − H_2_O + H]^+^ (calcd. for C_9_H_15_O_2_^+^: 155.1072), giving a molecular formula C_9_H_16_O_3_ to **4**. Corresponding to the ^13^C NMR and HRMS, there are two more protons in the ^1^H NMR compared with **3**. According to the detailed 2D NMR analysis (Figure 2), the planar structure of **4** was established as shown in Figure 1. The stereochemistry of C-4 was assumed to be the same as that of 3 based on the biogenetic and biosynthetic viewpoint, whereas the stereochemistry of C-7 remained unknown currently. The *cis* geometric configuration of the double bond Δ^5,6^ was determined by the peak pattern of the olefinic protons H-5/H-6 which overlapped to form what looks like a broad singlet with coupling constant of *J*_5,6_ obviously less than 15 Hz. Compound **4**, therefore, was elucidated to be (4*S*,*Z*)-7-hydroxy-4-methyloct-5-enoic acid, trivially named gliomacid B.

All the isolates were evaluated for cytotoxic activity against the proliferation of HeLa cell line and the antifouling effect against the settlement of *Balanus amphitrite* larvae. Unfortunately, none of the tested compounds **1**–**4** showed cytotoxicity at the concentrations up to 25 μg/mL. Pleasingly, compound **1** exhibited a non-toxic moderate inhibitory effect against the settlement of *Balanus amphitrite* larvae with IC_50_ being 12.8 μg/mL and LC_50_ > 25 μg/mL, whereas **2**–**4** displayed no anti-fouling effect at the concentrations up to 25 μg/mL.

## 3. Experimental Section

### 3.1. General Experimental Procedures

Optical rotation was measured on a JASCO P-2000 digital polarimeter (Jasco, Tokyo, Japan). The ultraviolet (UV) spectrum was recorded on a Shimadzu UV2401PC spectrophotometer (Shimadzu, Kyoto, Japan). IR spectra were recorded on a Perkin-Elmer 16 PC Fourier transform–infrared (FT–IR) spectrometer (Perkin-Elmer, Boston, MA, USA). NMR spectra were recorded on Varian Inova-500 NMR spectrometer (Toso, Tokyo, Japan) and Varian Mercury VX 300 spectrometer (Varian, Japan) using TMS (Tetramethylsilane) as internal standard, the chemical shifts were reported in ppm relative to CD_3_OD (δ_H_ 3.31 and δ_C_ 49.00) or CDCl_3_ (δ_H_ 7.26 and δ_C_ 77.16). High-resolution mass spectra (HRMS) were performed on QSTAR XL mass spectrometry system (Applied Biosystems, Foster City, CA. USA). The preparative HPLC was performed on Waters systems (Waters, Manchester, UK) involving Waters 2545 binary gradient module, Waters 2996 PDA detector, MassLynx V4.1 workstation, and XBridge RP18 column (19 × 300 mm, 5 µm). Column chromatography (CC) was performed with silica gel (230–400 mesh, Merck, Darmstadt, Hessen, Germany) or octadecyl silane (ODS) (Fuji Silysia Chemical Ltd. Kasugai, Aichi, Japan). TLC was performed on aluminium sheet precoated with silica gel G_60_ (Merck, Darmstadt, Hessen, Germany). Spots were visualized under UV light or by spraying with 5% H_2_SO_4_ in EtOH followed by heating.

### 3.2. Fungal Material

The fungal strain, *Gliomastix* sp. ZSDS1-F7-2, was isolated from the sponge *Phakellia fusca* Thiele collected from the Xisha Islands of China in 2012. The sponge was identified by Prof. Jinhe Li at the Institute of Oceanology, Chinese Academy of Sciences. A voucher specimen of the fungus (No. 12071301) was deposited in the Key Laboratory of Tropical Marine Bio-resources and Ecology, South China Sea Institute of Oceanology, Chinese Academy of Sciences. The fungus was identified using a molecular biological protocol by DNA amplification and sequencing of the 18S rRNA gene (GenBank Accession No. KR054959). By comparison with the GenBank database, the most similar strain was *Gliomastix murorum* YNS1116-4, with a sequence identity of 99% (95% query coverage). So the fungus strain ZSDS1-F7-2 belonged to the genus *Gliomastix*, and was designated as *Gliomastix* sp.

### 3.3. Fermentation, Extraction and Isolation

The strain *Gliomastix* sp. ZSDS1-F7-2 was cultured in 100 mL Erlenmeyer flasks each containing 25 mL of malt medium (20 g/L of malt extract (Oxoid), 1 g/L of peptone (BD Difco), and 30 g/L of sea salt (Anhui Hongsifang Co., Ltd., CNSG, Anhui, China), to give the seed culture (20 × 100 mL flasks), which was transferred to the 1000 mL Erlenmeyer flask containing solid rice medium (200 g of rice (North-eastern rice, COFCO), 200 g water, 6 g sea salt (Anhui Hongsifang Co., Ltd., CNSG, Anhui, China, 20 × 1 L flasks). After incubation at 25 °C under daylight for 2 months, the culture was broken up with a spatula and extracted with 95% EtOH (3 × 10 L). The combined extract was filtered and evaporated under vacuum to give 95% EtOH extract (105.1 g), which was suspended in water (1 L) and extracted successively with petroleum ether (1 L × 3), EtOAc (1 L × 3), and *n*-BuOH (1 L × 3). The EtOAc fraction (70 g) was subjected to a chromatography column (CC) on silica gel (400 g; 9.0 cm diam × 50 cm height), eluted with a step gradient of petroleum ether (PE) in EtOAc (10–100%), and then with MeOH–EtOAc (10–30%), to yield 10 fractions (Fr. A-J). Fr. B (5.0 g) was further subjected to octadecyl silane (ODS) CC (60 g; 3.0 cm diam. × 16 cm height), eluted with a gradient solution of methanol in water (10–100%) to furnish 10 fractions (Fr. B1-Fr. B10). Then, Fr. B4 (0.2 g) was purified by preparative high-performance liquid chromatography (HPLC) (25% acetonitrile–H_2_O, 15.0 mL/min) to yield compounds **1** (19.5 mg), **2** (5.3 mg). Fr. B6 (0.3 g) was purified by preparative HPLC (30% acetonitrile-H_2_O, 15.0 mL/min) to yield a mixture (18.9 mg) containing both compounds **3** and **4**. To the solution of this mixture (15.0 mg) in dried DMF (1 mL), was added K_2_CO_3_ (40 mg), followed by benzyl bromide (16 μL), then this was stirred at room temperature for 8 h, then quenched with saturated NaHCO_3_ aqueous (2 mL), extracted with DCM (3 mL × 2), washed sequentially by brine (5 mL × 2) and water (5 mL); the residue obtained after evaporation was purified by silica gel CC, eluting with 15% EtOAc/PE, to give the benzyl ester of **3** (6.5 mg, ^1^H NMR recorded at 300 MHz in CDCl_3_: δ_H_ 1.06 (3H, d, *J* = 6.9), 1.70 (3H, d, *J* = 6.3), 2.52 (1H, m), 4.01 (1H, m), 5.18 (2H, s), 5.47 (1H, m), 5.70 (1H, m), 5.90 (1H, d, *J* = 15.9), 7.03 (1H, dd, *J* = 15.9, 6.9), 7.38 (5H, m)), and the benzyl ester of 4 (3.0 mg, 1H NMR recorded at 300 MHz in CDCl3: δH 0.99 (3H, d, *J* = 6.6), 1.24 (3H, d, *J* = 6.3), 1.66 (2H, m), 2.13 (1H, m), 2.34 (2H, t, *J* = 7.5), 4.23 (1H, m), 5.11 (2H, s), 5.46 (2H, m), 7.36 (5H, m)). The separated benzyl esters **3** (3.0 mg), was dissolved in methanol/water (1 mL/0.5 mL), followed by the addition of LiOH (2.1 mg), the mixture was stirred at room temperature for 4 h, then quenched with 1 M HCl aqueous (2 mL), extracted with DCM (3 mL × 2), washed with brine (3 mL × 2) and water (3 mL), and the combined DCM layer was evaporated to obtain the residue which was subjected to CC, eluted with 30% EtOAc/PE to give **3** (2.0 mg). The compound **4** (1.2 mg) was obtained from the separated benzyl esters **4** by the same procedure.

Gliomasolide F (**1**): brown oil; ${\left[\alpha \right]}_{D}^{25}$ +88.1 (*c* = 5.53, CH_3_OH); IR (film) ν_max_ 3487, 2938, 1746, 1450, 1377, 1111, 1027, 820 cm^−1^; UV (CH_3_OH) λ_max_ (log *ε*) 210 (3.75) nm; CD λ_max_ (Δ*ε*) 260 (18.9); ^1^H and ^13^C NMR data see Table 1; HR-TOF-MS *m*/*z* 169.0872 [M + H]^+^ (calcd. for C_9_H_13_O_3_^+^: 169.0865).

Gliomasolide G (**2**): brown oil; ${\left[\alpha \right]}_{D}^{25}$ −38.7 (*c* = 0.87, CH_3_OH); IR (film) ν_max_: 3503, 2966, 1718, 1459, 1249, 1051, 966, 844 cm^−1^; UV (CH_3_OH) λ_max_ (log *ε*): 244 (3.32) nm; ^1^H and ^13^C NMR data see Table 1; HR-TOF-MS *m*/*z* 171.1029 [M + H]^+^ (calcd. for C_9_H_15_O_3_^+^: 171.1021), 153.0932 [M − H_2_O + H]^+^ (calcd. for C_9_H_13_O_2_^+^: 153.0916).

Gliomacid A (**3**): brown oil; ${\left[\alpha \right]}_{D}^{25}$−409.7 (*c* 0.08, MeOH); IR (film) ν_max_ 3394, 2917, 1697, 1652, 1398, 967 cm^–1^; UV (CH_3_OH) *λ*_max_(log *ε*) 232 (3.86) nm; ^1^H and ^13^C NMR data see Table 2; HR-TOF-MS *m*/*z* 153.0924 [M − H_2_O + H]^+^ (calcd. for C_9_H_13_O_2_^+^: 153.0916).

Gliomacid B (**4**): brown oil; ${\left[\alpha \right]}_{D}^{25}$ +76.8 (*c* 0.09, MeOH); IR (film) ν_max_ 3382, 2923, 1707, 1651, 1379, 1059 cm^–1^; UV (CH_3_OH) *λ*_max_(log *ε*) 228 (2.95) nm; ^1^H and ^13^C NMR data see Table 2; HR-TOF-MS *m*/*z* 155.1073 [M − H_2_O + H]^+^ (calcd. for C_9_H_15_O_2_^+^: 155.1072).

### 3.4. Computation Section

Conformational studies were performed using the Amber and MMFF94S force field, respectively. The geometries with relative energy less than 5.5 kcal/mol were selected and calculated at B3LYP/6-31G(d)//B3LYP/3-21G(d) level to obtain global minima. Conformers within an energy range of 3 kcal/mol from the global minima were subjected to geometrical optimization (DFT/B3LYP/6-31G(d)) in the gas phase, combined with calculation of vibrational modes to confirm these minima. No imaginary frequencies were found. After conformational search, 10 conformations for (4*R*, 5*S*)-**1**, and 8 conformations for (4*R*, 5*R*)-**1** with low energy were found. For these conformations, ECD spectra were calculated at the B3LYP/6-311+G(d,p) level in MeOH (SCRF/IEFPCM). OR values were calculated at the B3LYP/6-311++G(2d,p) level. Each calculated ECD spectra and OR values was assigned a Boltzmann weight according to the energy of the minimized conformers at 298.15 K and overlaid prior to comparison with the experimental results. All of the DFT (density functional theory) calculations reported in this study were performed with the Gaussian 09 package [21].

### 3.5. Cytotoxicity Test

The cytotoxicity against HeLa cervical cancer cell line was determined using the MTT [3-(4,5-dimethylthiazol-2-yl)-2,5-diphenyltetrazolium bromide] method. Briefly, the HeLa cells were cultured in Dulbecco’s modified Eagle's medium (DEME) (Invitrogen, Carlsbad, CA, USA) supplemented with 10% FCS (fetal calf serum) (Invitrogen, Carlsbad, CA, USA) in a 96-well plate at a density of 1 × 10^4^ cells per well and incubated for 20 hours, then the cells were treated with testing samples at various concentrations and then incubated at 37 °C for 48 hours in a 5% CO_2_ atmosphere. The supernatant was removed, followed by the addition of 25 μL of MTT (2.5 mg mL^−1^ in PBS (phosphate buffered saline)) to each well. After incubation at 37 °C for 4 h, 100 μL of dimethyl sulfoxide (DMSO) was added to each well and incubated for another 20 min. The absorbance of each well was then measured at 570 nm using a Thermo scientific Multiskan FC multiplate photometer (Waltham, MA, USA).

### 3.6. Antifouling Effect

The antifouling assay was evaluated according to the procedure reported previously [13]. Briefly, fresh cyprids of the barnacle *Balanus amphitrite* were used in the testing. Larval settlement assays were examined using 24-well polystyrene plates with Sea-Nine 211^TM^ (4,5-dichloro-2-*n*-octyl-4-iso-thiazolin-3-one) being served as positive control. The tested samples and Sea-Nine 211^TM^ were dissolved with a small amount of dimethyl sulfoxide (DMSO) and then diluted with 0.22 μm filtered seawater (FSW) to achieve the final concentrations of 25.0, 12.5, 6.25, 3.13, and 1.56 µg/mL. About 15−20 competent larvae were added to each well containing 1 mL of test solution in triplicate, with wells having only FSW, DMSO, and larvae being served as negative control. The plates were incubated at 25 °C for 48 h, followed by examination under a microscope to count settled, unsettled larvae, and where appropriate, potential toxic effects were recorded. The number of settled larvae was expressed as a percentage of the total number of larvae per well. The EC_50_ was calculated as the concentration where 50% of the larval individuals were inhibited to settle as compared to the control, while LC_50_ was calculated as the concentration where 50% of the larval population was dead.

## 4. Conclusions

As part of our continuous effort to explore the biologically and structurally interesting metabolites from marine resources, we carried out a systematical investigation on the secondary metabolites from the sponge-associated fungus *Gliomastix* sp. ZSDS1-F7-2, leading to the discovery of four new structurally related metabolites, each having nine carbons in total, therefore briefly named as C9 metabolites. Their structures involving absolute configurations were successfully elucidated by the detailed analyses of NMR, HRMS, quantum chemistry calculations, biosynthetic relationship, and by comparison with reported literature. Biological assay revealed that these C9 metabolites showed no cytotoxic effect against the HeLa cell line, whereas one γ-lactone named gliomasolide F (1) exhibited moderate anti-fouling activity against the settlement of the barnacle *Balanus Amphitrite.* Furthermore, based on their structure similarity, a plausible biosynthetic relationship between these C9 metabolites was assumed. Interestingly, the co-isolation of unusual macrolides gliomasolides A—E and these four look like simple C9 metabolites, from the same fermentation extract of the marine fungus *Gliomastix* sp. ZSDS1-F7-2, and strongly inspired our assumption that these C9 metabolites might represent the early steps of the polyketide pathway toward gliomasolides A—E or other macrolides that remained unknown within the fermentation extract. For a more detailed understanding of the role of these C9 metabolites in the macrolides biosynthesis, incorporation experiments will be carried out in our laboratory in the future.

## Figures and Tables

**Table 1:** ^1^H (300 MHz) and ^13^C (75 MHz) nuclear magnetic resonance (NMR) data of compounds **1** in CDCl_3_ and **2** in CD_3_OD.

No.	1		2	
	δ_H_ (Mult., *J* in Hz)	δ_C_	δ_H_ (Mult., *J* in Hz)	δ_C_
1	-	172.8	-	173.4
2	6.05 (d, *J* = 5.4)	121.8	2.42 (dd, *J* = 18.3, 9.0) 2.81 (dd, *J* = 18.3, 6.6)	36.4
3	7.40 (d, *J* = 5.4)	158.5	4.20 (m)	67.4
4	-	90.9	2.16 (m)	38.9
5	4.14 (d, *J* = 6.9)	75.8	4.81 (dd, *J* = 6.9, 2.1)	83.2
6	5.43 (dd, *J* = 15.0, 6.9)	127.6	5.66 (dd, *J* = 15.3, 6.9)	128.6
7	5.78 (dq, *J* = 15.0, 6.0)	131.0	5.84 (dq, *J* = 15.3, 6.3)	130.6
8	1.70 (d, *J* = 6.0)	17.9	1.75 (d, *J* = 6.3)	17.9
9	1.40 (s)	20.1	0.92 (d, *J* = 7.2)	5.9

**Table 2:** ^1^H (500 MHz) and ^13^C (75 MHz) NMR data of compounds **3**–**4** in CD_3_OD.

No	3		4	
	δ_H_ (Mult., *J* in Hz)	δ_C_	δ_H_ (Mult., *J* in Hz)	δ_C_
1	-	171.2	-	179.1
2	5.79 (d, *J* = 15.5)	123.7	2.25 (m)	33.9
3	6.89 (dd, *J* = 15.5,7.5)	151.5	1.57 (m), 1.62 (m)	33.4
4	2.43 (m)	43.9	2.13 (m)	37.4
5	3.93 (m)	76.9	5.47 (m)	136.2
6	5.44 (dd, *J* = 15.5, 7.5)	132.8	5.47 (m)	134.8
7	5.68 (dq, *J* = 15.5, 6.5)	128.8	4.18 (m)	69.3
8	1.70 (d, *J* = 6.5)	17.9	1.20 (d, *J* = 6.5)	23.7
9	1.06 (d, *J* = 7.0)	15.4	1.01 (d, *J* = 6.5)	20.9

## Supplementary Materials

The following are available online at http://www.mdpi.com/1660-3397/16/7/231/s1, Table S1: ^1^H and ^13^C NMR data of compounds **2** and prelactone C; Computational data, HRMS, IR and NMR spectra of compounds **1**–**4**.
