# Supplementary material for: Four New C9 Metabolites from the Sponge-Associated Fungus Gliomastix sp. ZSDS1-F7-2
Source: Mar Drugs. 2018 Jul 9;16(7):231. doi: 10.3390/md16070231 (PMC6071072; doi:10.3390/md16070231)
Supplement: Supplementary file 1 [file marinedrugs-16-00231-s001.pdf]

Supporting information for journal *Marine drugs*

Four New C9 Metabolites from the Sponge-associated Fungus *Gliomastix* sp. ZSDS1-F7-2

Jun Zhang [1,2,*], Zhiqiang Yang [1], Yan Liang [1], Liping Zhong [1], Huiting Lin [1], Balian Zhong [1], Liangchun Li [4,*], Shihai Xu [2,*] and Yonghong Liu [3]

[1] National Engineering Research Center of Navel Orange, Gannan Normal University, Ganzhou, 341000, China; 451058969@qq.com (Z.Y.); 76675104@qq.com (Y.L.); 2280648398@qq.com (L.Z.); 1042143321@qq.com (H.L.); bal.zh@163.com (B.Z.)

[2] Department of Chemistry, Jinan University, Guangzhou, 510000, China.

[3] Center for Marine Microbiology, South China Sea Institute of Oceanology, Chinese Academy of Sciences, Guangzhou 510000, China; yonghongliu@scsio.ac.cn (Y.H.L.)

[4] School of Life Science and Engineering, Southwest University of Science and Technology, Mianyang, 621010, China.

***** Correspondence: bri71527152@outlook.com (J.Z.); txush@jnu.edu.cn (S.X.); lilc76@gmail.com (L.L.); Tel.: +86-797-8393068 (J.Z.); +86-20-85221346 (S.X.); +86-816-6089531 (L.L.)

Table of contends

**Table S1.** ^{1}H and ^{13}C NMR data of compounds **2** and prelactone C	Page 3	**Figure S20.** ^{1}H NMR (500 MHz, CD_3OD) of compound **4**.	Page 23
**Table S2.** Anti-fouling and cytotoxicity activities of compounds **1–4**	3	**Figure S21.** ^{13}C NMR (75 MHz, CD_3OD) of compound **4**	24
**Figure S1.** The conformers, relative energies (ΔG) and conformer populations of the minimized conformers of (4*R*, 5*S*)**-1** within an energy range of 3 kcal/mol.	4	**Figure S22.** ^{1}H-^{1}H COSY (500 MHz, CD_3OD) of compound **4**	25
**Figure S2**. The conformers, relative energies (ΔG) and conformer populations of the minimized conformers of (4*R*, 5*R*)**-1** within an energy range of 3 kcal/mol.	5	**Figure S23.** HSQC (500 MHz, CD_3OD) of compound **4**.	26
**Figure S3.** ^{1}H NMR (300 MHz, $CDCl_3$) of compound **1**	6	**Figure S24.** HMBC (500 MHz, CD_3OD) of compound **4**.	27
**Figure S4.** ^{13}C NMR (75 MHz, $CDCl_3$) of compound **1**	7	**Figure S25**. HRMS of compound **1**.	28
**Figure S5.** ^{1}H-^{1}H COSY (300 MHz, $CDCl_3$) of compound **1**	8	**Figure S26.** HRMS of compound **2**.	29
**Figure S6.** HSQC (300 MHz, $CDCl_3$) of compound **1**.	9	**Figure S27.** HRMS of compound **3**.	30
**Figure S7.** HMBC (300 MHz, $CDCl_3$) of compound **1**.	10	**Figure S28.** HRMS of compound **4**.	31
**Figure S8.** ^{1}H NMR (300 MHz, CD_3OD) of compound **2**.	11	**Figure S29**. IR of compound **1**.	32
**Figure S9.** ^{13}C NMR (75 MHz, CD_3OD) of compound **2**.	12	**Figure S30**. IR of compound **2**.	33
**Figure S10.** ^{1}H-^{1}H COSY (300 MHz, CD_3OD)compound **2**	13	**Figure S31**. IR of compound **3**.	34
**Figure S11.** HSQC (300 MHz, CD_3OD) of compound **2**.	14	**Figure S32**. IR of compound **4**.	35
**Figure S12.** HMBC (300 MHz, CD_3OD) of compound **2**.	15		
**Figure S13.** NOESY (300 MHz, CD_3OD) of compound **2**.	16		
**Figure S14.** ^{1}H NMR (300 MHz, CD_3OD) of compounds **3** and **4**.	17		
**Figure S15.** ^{1}H NMR (500 MHz, CD_3OD) of compound **3**.	18		
**Figure S16.** ^{13}C NMR (75 MHz, CD_3OD) of compound **3**	19		
**Figure S17.** ^{1}H-^{1}H COSY (500 MHz, CD_3OD) of compound **3**	20		
**Figure S18.** HSQC (500 MHz, CD_3OD) of compound **3**.	21		
**Figure S19.** HMBC (500 MHz, CD_3OD) of compound **3**.	22		

Table S1. ^{1}H and ^{13}C NMR data of compounds **2** and **prelactone C**[d]

	**2**[a]		**prelactone C**[b]	**prelactone C**[c]	
No	δ_H (mult., J in Hz)	δ_C	δ_H (mult., J in Hz)	δ_H (mult., J in Hz)	δ_C
1	-	173.4	-	-	170.2
2	2.42 (dd, J = 18.3, 9.0)<br>2.81 (dd, J = 18.3, 6.6)	36.4	2.40 (dd, J = 17, 7)<br>2.91 (dd, J = 17, 6)	2.50 (dd, J = 17, 8)<br>2.94 (dd, J = 17, 6)	39.1
3	4.20 (m)	67.4	3.72 (ddq, J = 7,7,6)	3.79 (m)	69.6
4	2.16 (m)	38.9	1.60 (ddq, J = 10, 7, 6)	1.68 (m)	41.5
5	4.81 (dd, J = 6.9, 2.1)	83.2	4.30 (dd, J = 10, 8)	4.21 (m)	84.1
6	5.66 (dd, J = 15.3, 6.9)	128.6	5.46 (ddq, J = 15, 8, 2)	5.46 (ddq, J = 15, 8, 2)	127.6
7	5.84 (dq, J = 15.3, 6.3)	130.6	5.83 (ddq, J = 15, 6, 1)	5.81 (ddq, J = 15, 6, 1)	132.6
8	1.75 (d, J = 6.3)	17.9	1.75 (dd, J = 6.5, 2)	1.76 (dd, J = 6.5, 2)	17.7
9	0.92 (d, J = 7.2)	5.9	1.04 (d, J = 6.5)	1.04 (d, J = 7.0)	13.7

[a]: Recorded for ^{1}H NMR at 300MHz/ ^{13}C NMR at 75MHz in CD_3OD; [b]: Recorded for ^{1}H NMR at 500MHz in CD_3OD; [c]: Recorded for ^{1}H NMR at 200MHz / ^{13}C NMR at 50MHz in $CDCl_3$; [d]: The NMR data of Prelactone C were taken from the literature, Bindseil, Kai U., and Axel Zeeck. "Metabolic products of microorganisms. Part 265. Prelactones C and B, oligoketides from Streptomyces producing concanamycins and bafilomycins." *Helvetica chimica acta* 76.1 (1993): 150-157.

Table S2. Anti-fouling and cytotoxicity activities of compounds **1**–**4**

**Compounds**	**Antifouling (*Balanus amphitrite*)**			**Cytotoxicity (HeLa)**
	EC_{50} (μg/mL)	LC_{50} (μg/mL)	LC_{50}/EC_{50}	IC_{50} (μg/mL)
**1**	12.8	>25	>1.9	>25
**2**	>25	>25	-	>25
**3**	>25	>25	-	>25
**4**	>25	>25	-	>25

- Not applicable

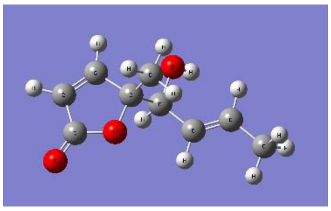 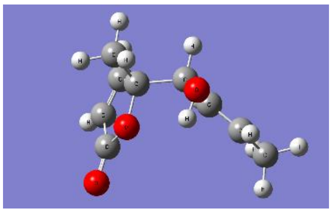 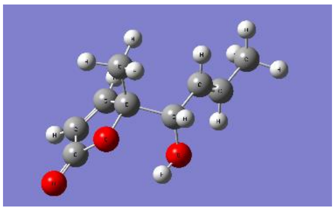

ΔG = 0 kcal/mol, 21%; ΔG = 0.14 kcal/mol, 17%; ΔG = 0.16 kcal/mol, 16%;

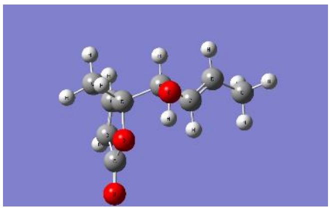 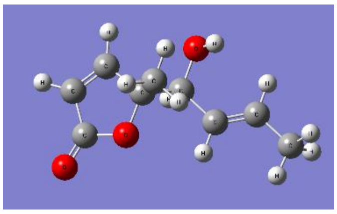 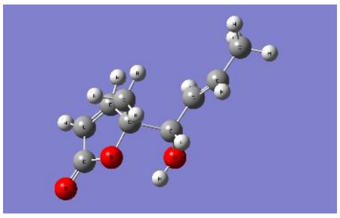

ΔG = 0.22 kcal/mol, 15%; ΔG = 0.36 kcal/mol, 11%; ΔG = 0.39 kcal/mol, 11%;

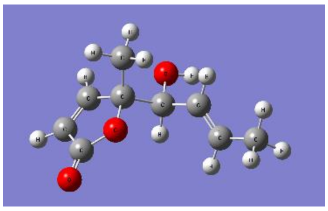 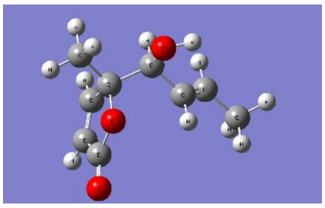 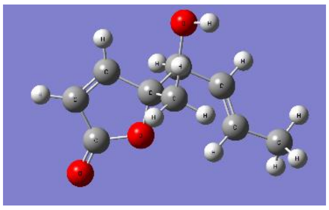

ΔG = 0.54 kcal/mol, 8%; ΔG = 2.00 kcal/mol, 0.7%; ΔG = 2.85 kcal/mol, 0.15%;

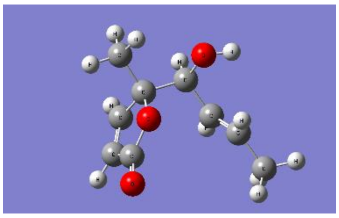

ΔG = 2.87 kcal/mol, 0.15%;

Figure S1. The conformers, relative energies (ΔG) and conformer populations of the minimized conformers of (4*R*, 5*S*)-**1** within an energy range of 3 kcal/mol.

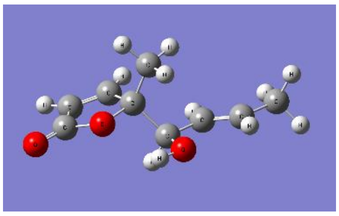 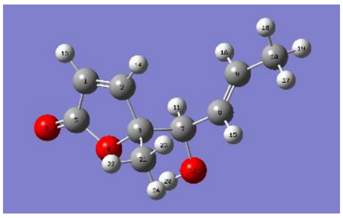 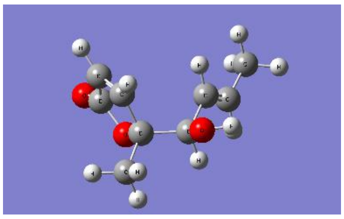

ΔG = 0 kcal/mol, 43%; ΔG = 0.09 kcal/mol, 37%; ΔG = 1.09 kcal/mol, 7%;

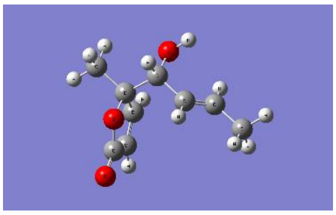 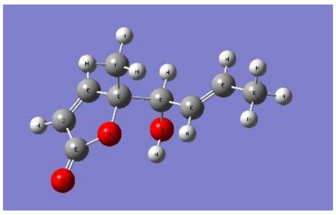 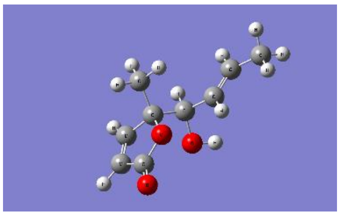

ΔG = 1.25 kcal/mol, 5%; ΔG = 1.41 kcal/mol, 4%; ΔG = 2.02 kcal/mol, 2%;

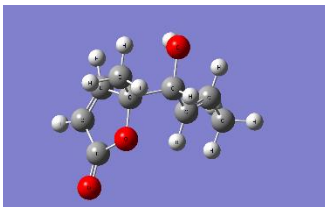 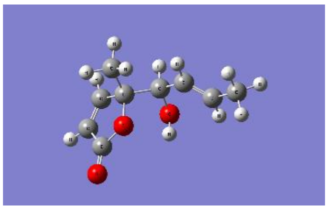

ΔG = 2.07 kcal/mol, 1%; ΔG = 2.31 kcal/mol, 1%.

Figure S2. The conformers, relative energies (ΔG) and conformer populations of the minimized conformers of (4*R*, 5*R*)-**1** within an energy range of 3 kcal/mol.

Figure S3. ^{1}H NMR (300 MHz, $CDCl_3$) of compound **1**.

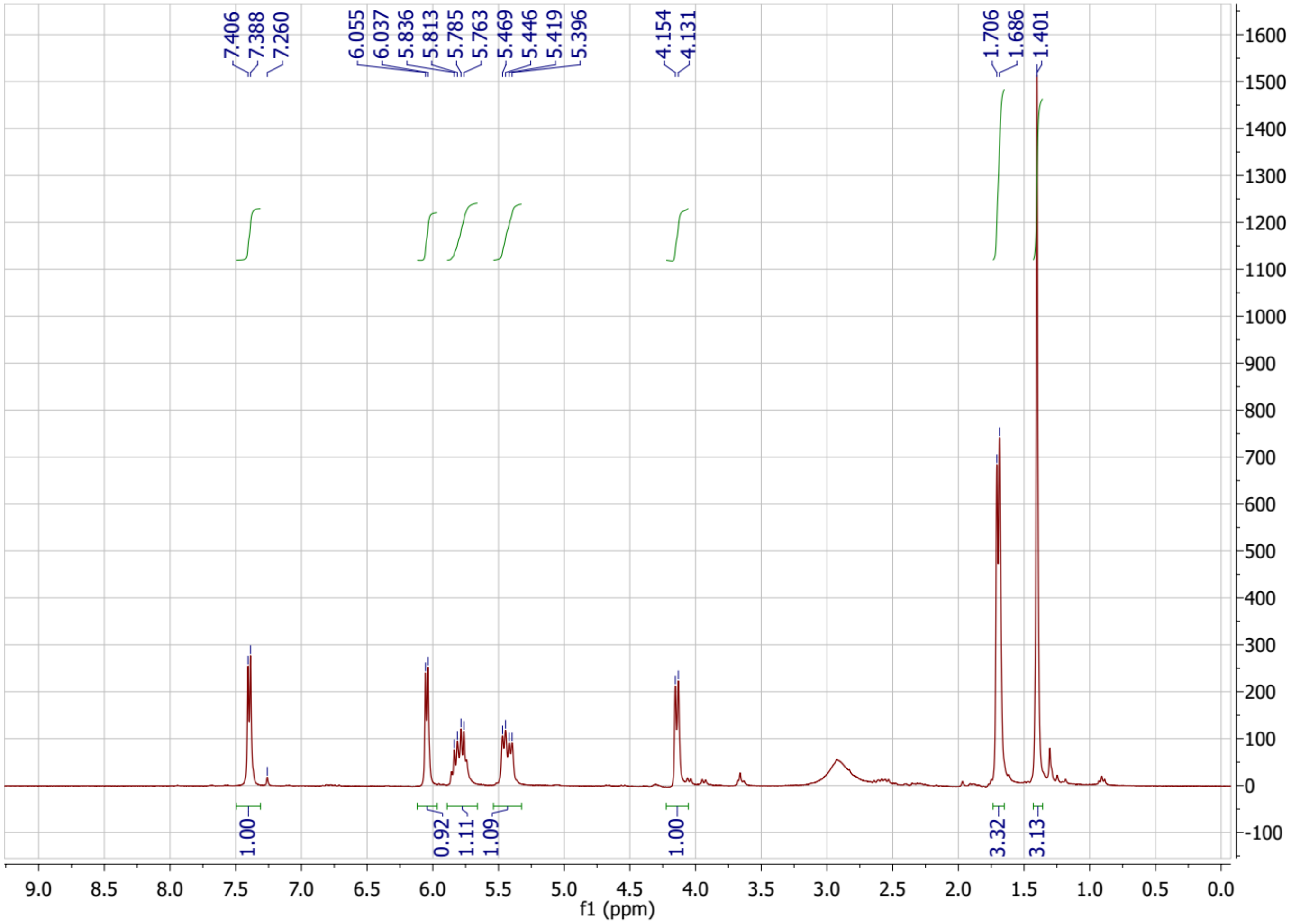

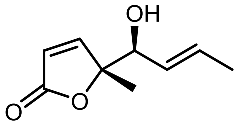

1

**Figure S4.** ^{13}C NMR (75 MHz, $CDCl_3$) of compound **1**.	
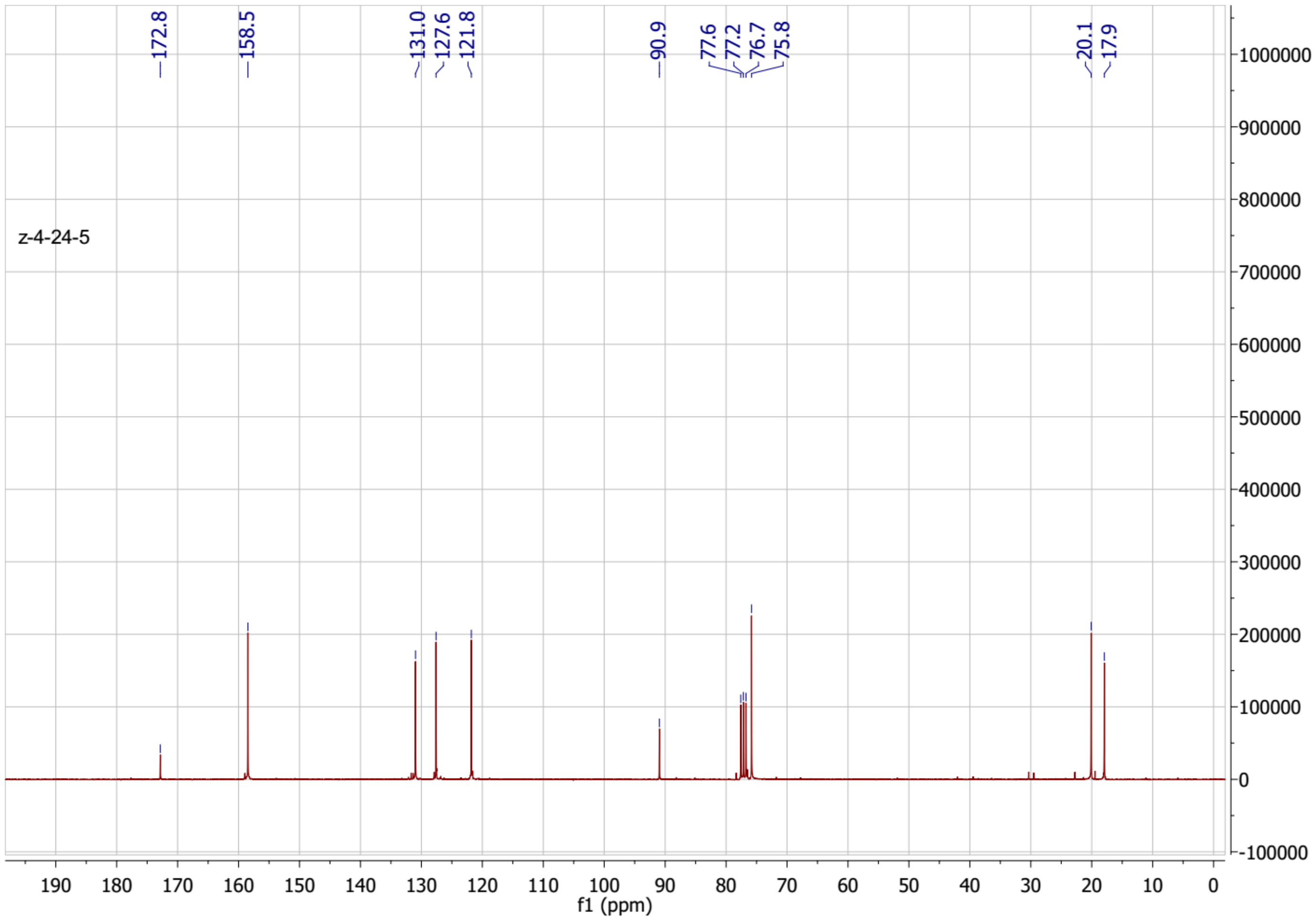	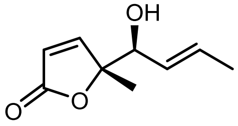 **1**

Figure S5. ^{1}H-^{1}H COSY (300 MHz, $CDCl_3$) of compound **1**

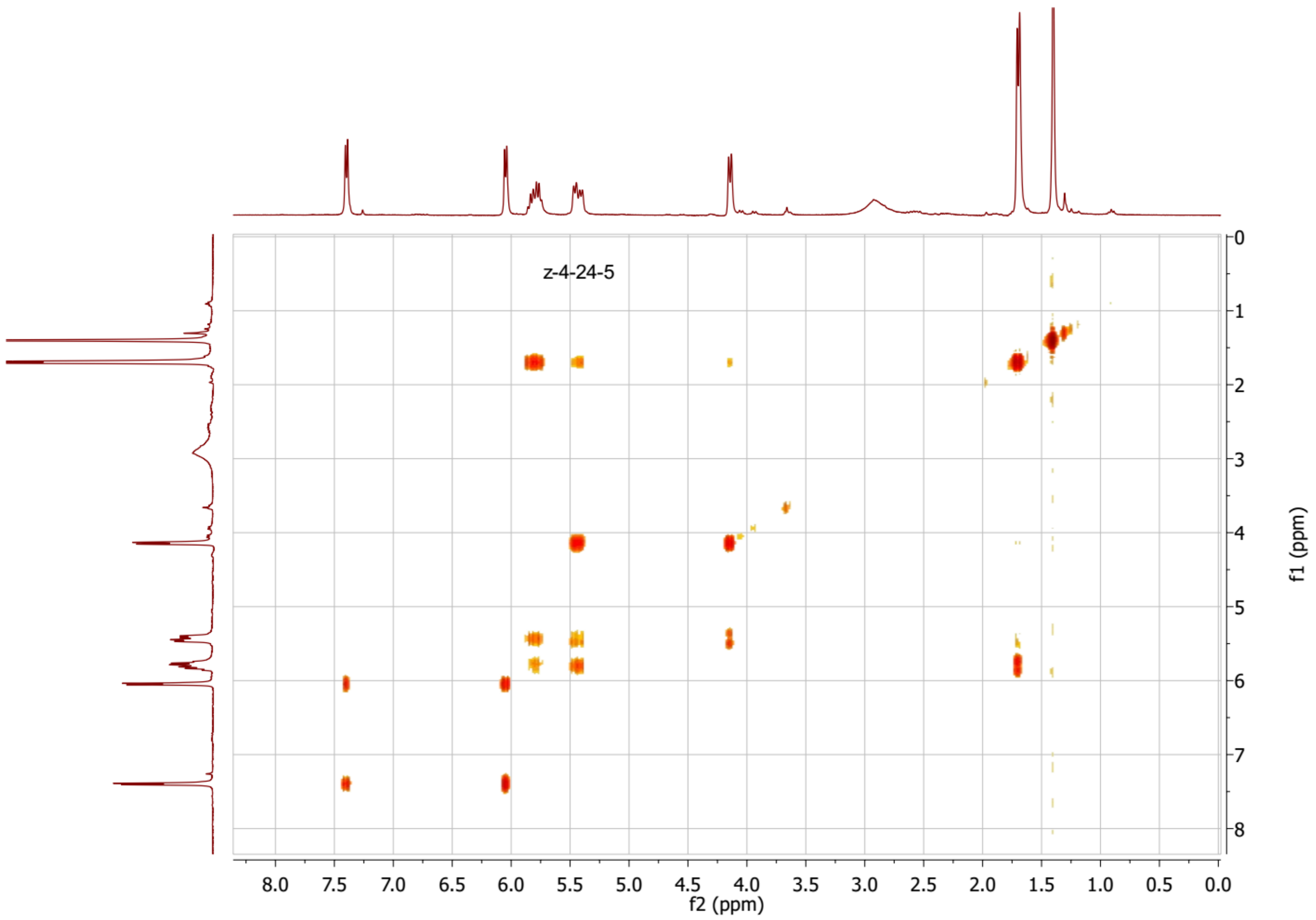

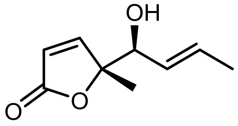

1

Figure S6. HSQC (300 MHz, $CDCl_3$) of compound **1**.

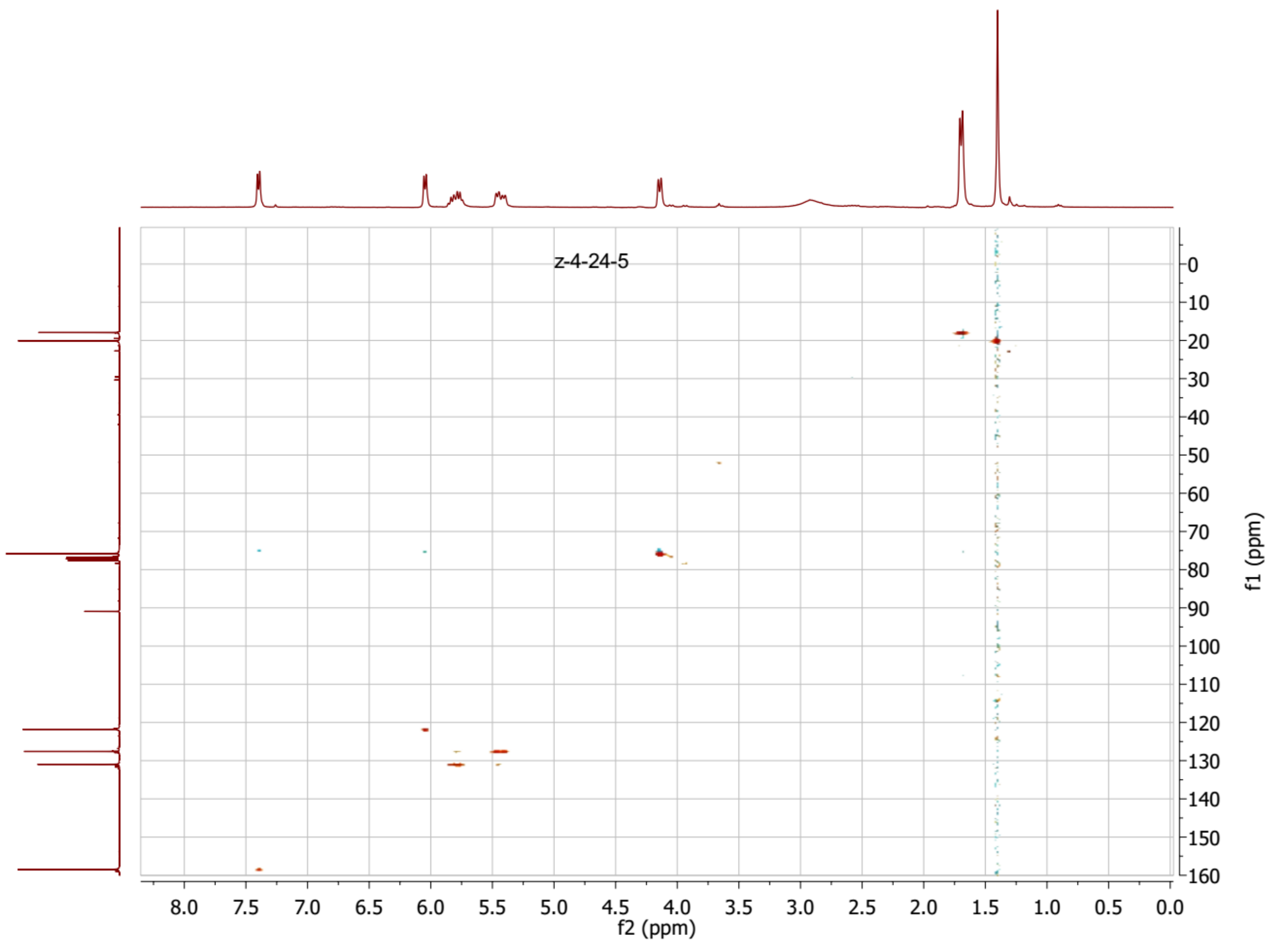

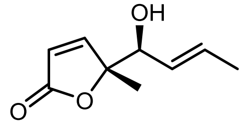

1

Figure S7. HMBC (300 MHz, $CDCl_3$) of compound **1**.

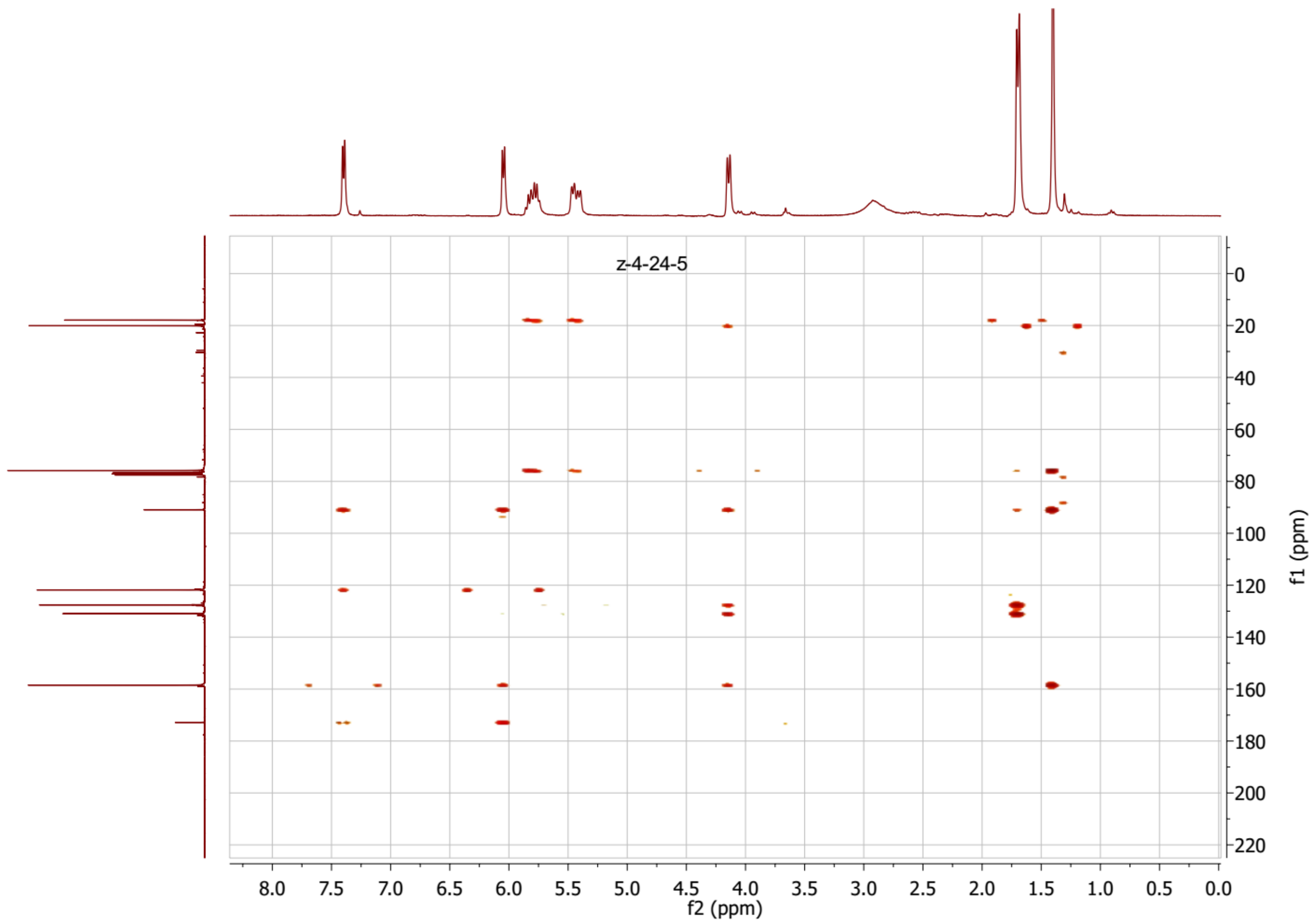

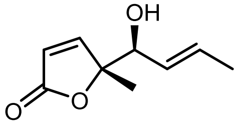

1

Figure S8. ^{1}H NMR (300 MHz, CD_3OD) of compound **2**.

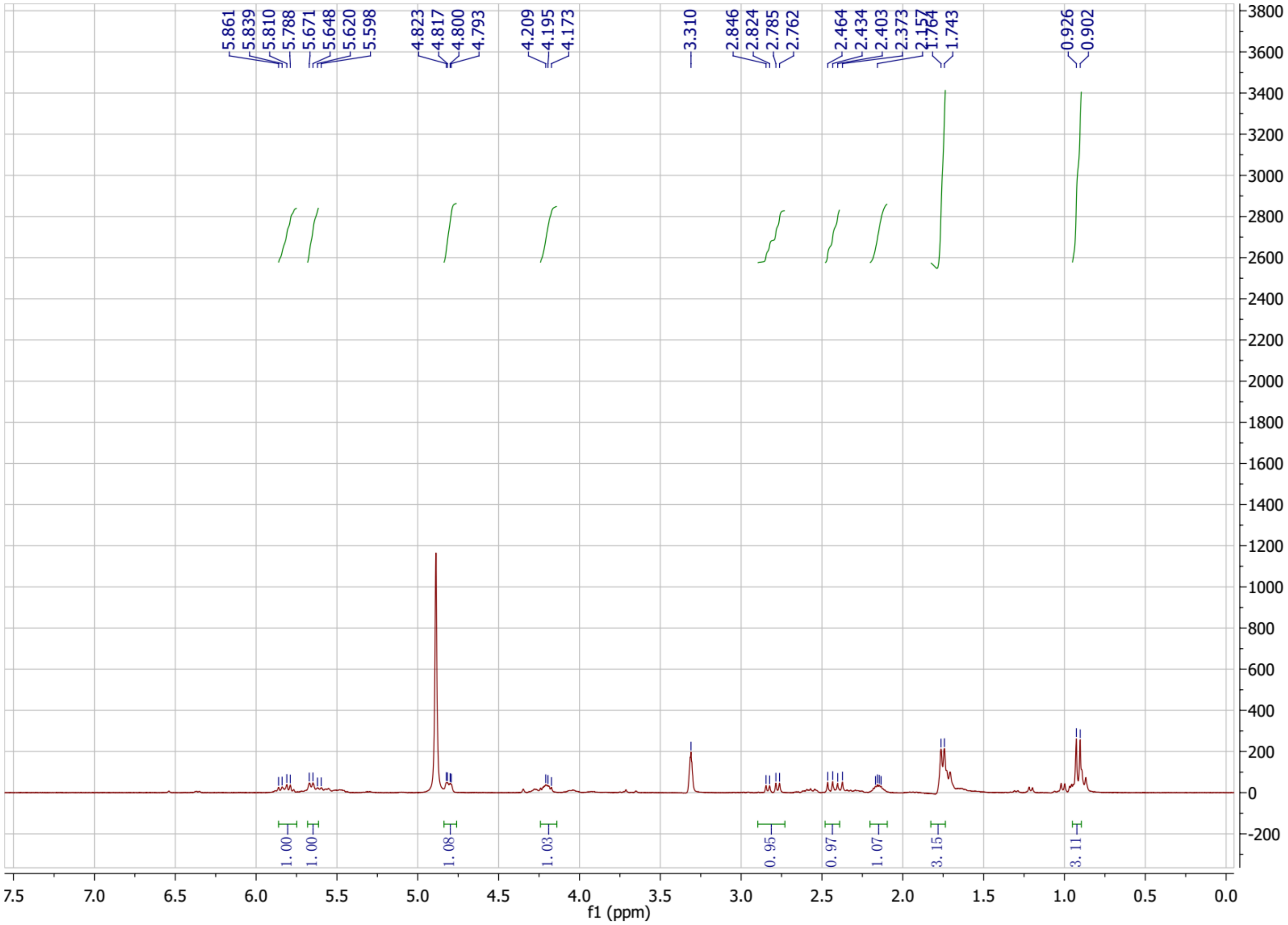

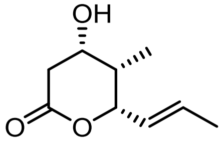

2

Figure S9. ^{13}C NMR (75 MHz, CD_3OD) of compound **2**.

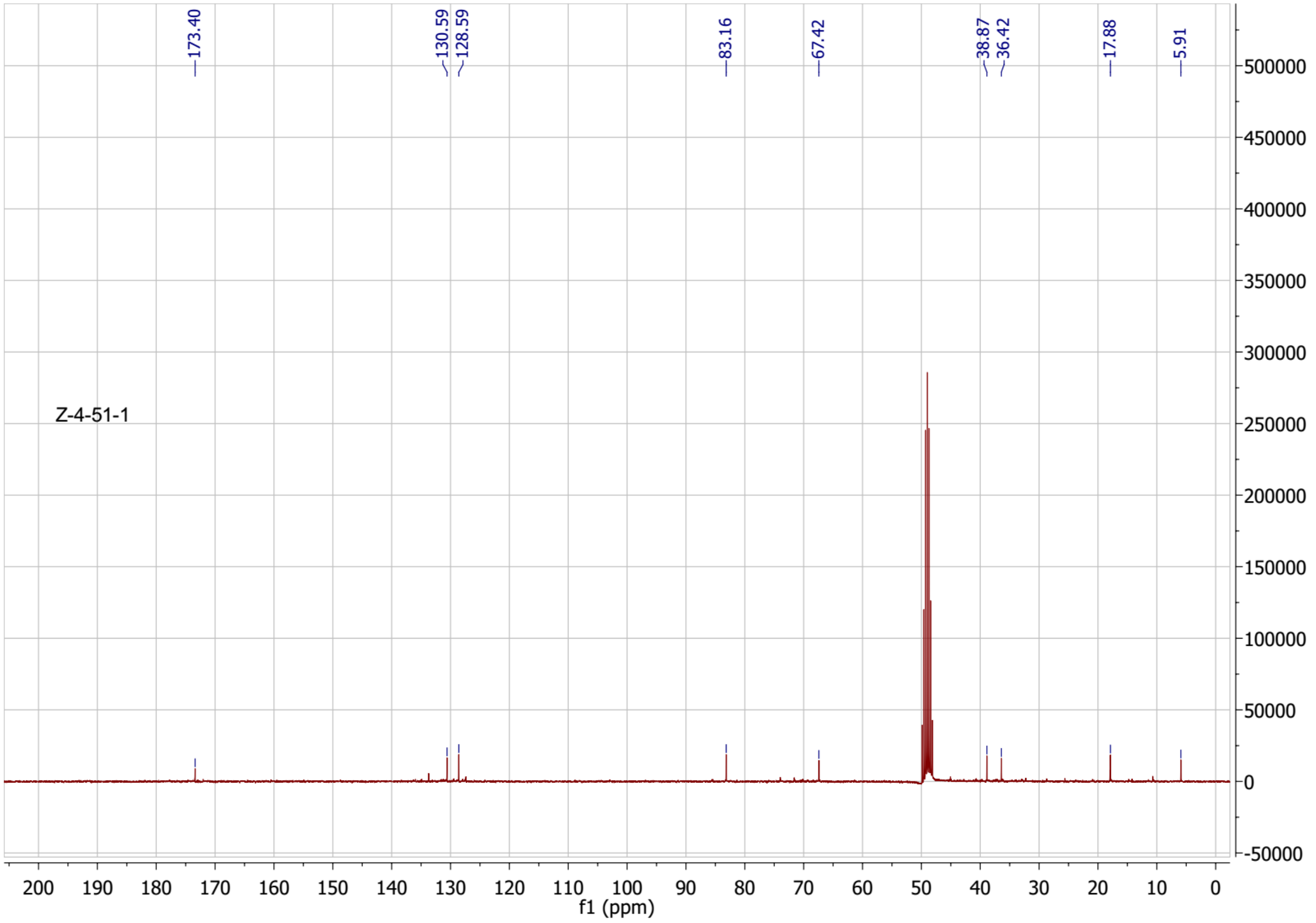

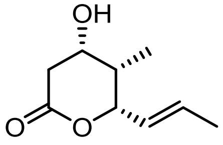

2

**Figure S10.** ^{1}H-^{1}H COSY (300 MHz, CD_3OD)compound **2**	
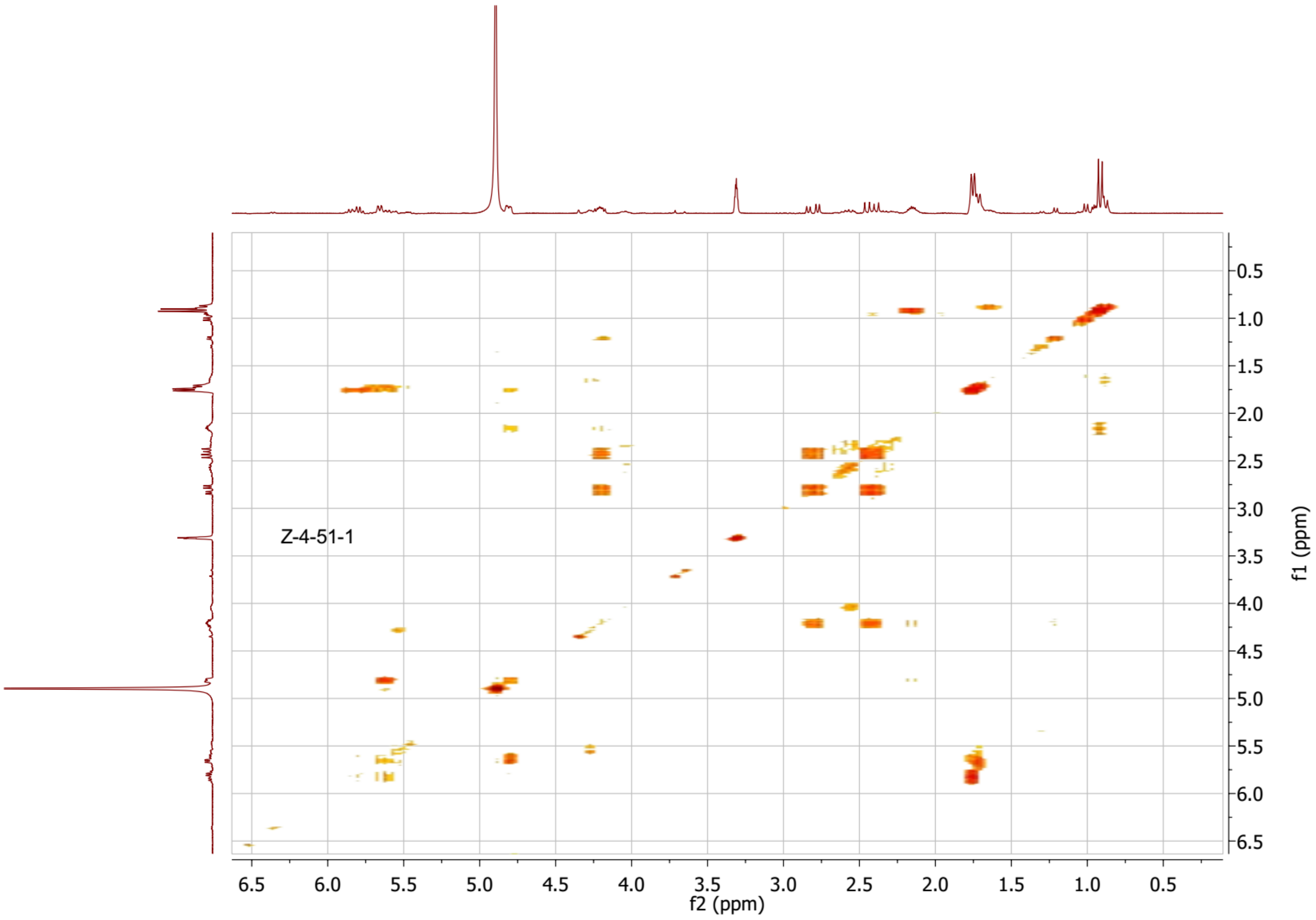	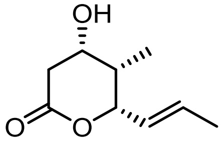**2**

Figure S11. HSQC (300 MHz, CD_3OD) of compound **2**.

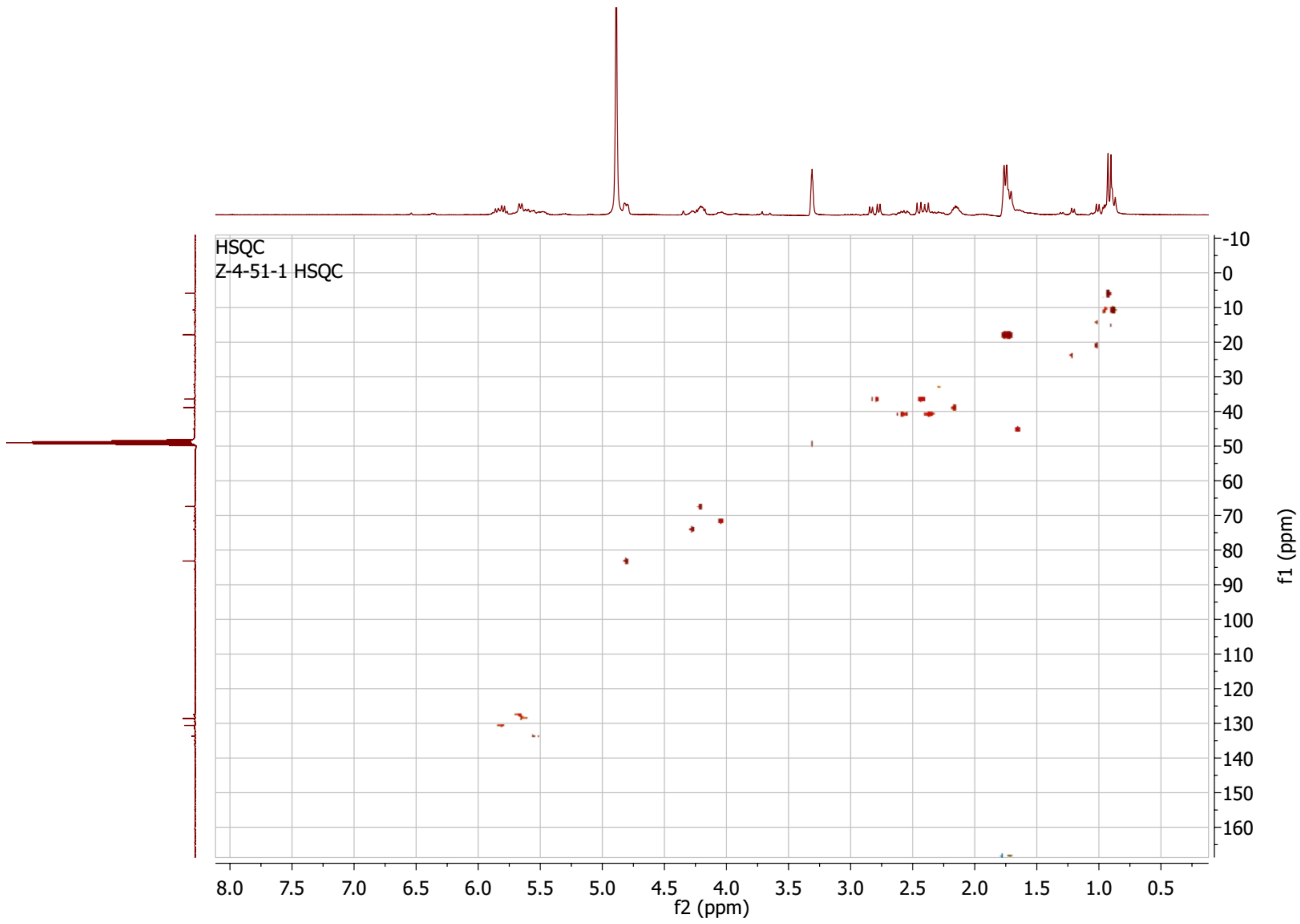

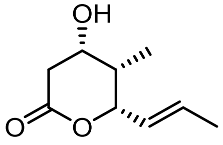

2

Figure S12. HMBC (300 MHz, CD_3OD) of compound **2**.

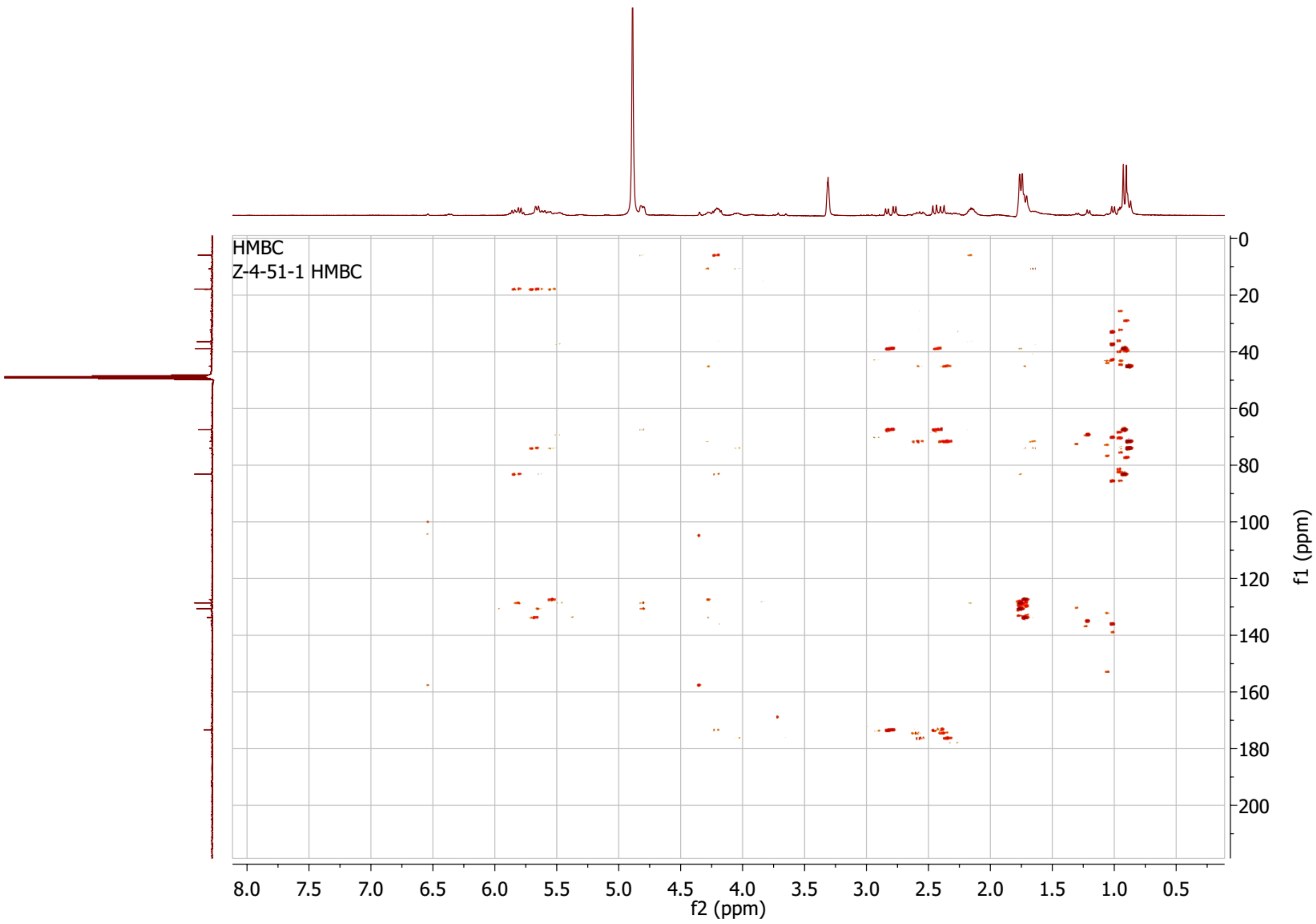

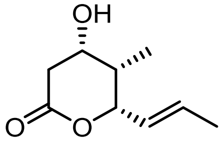

2

Figure S13. NOESY (300 MHz, CD3OD) of compound 2.	
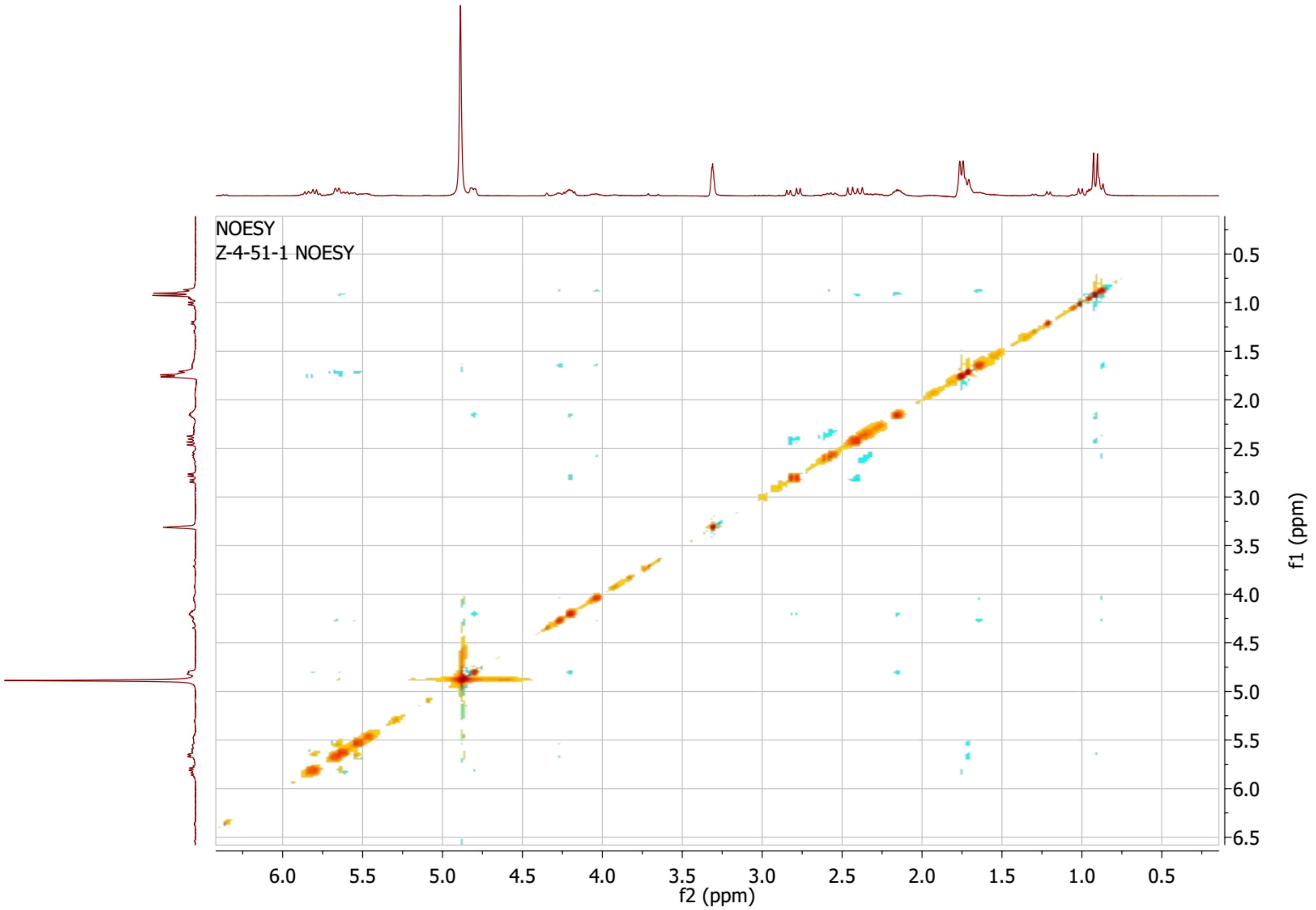	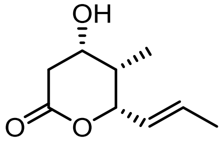<br>**2**

Figure S14. ^{1}H NMR (500 MHz, CD_3OD) of compounds **3** and **4** (1:1 ratio).

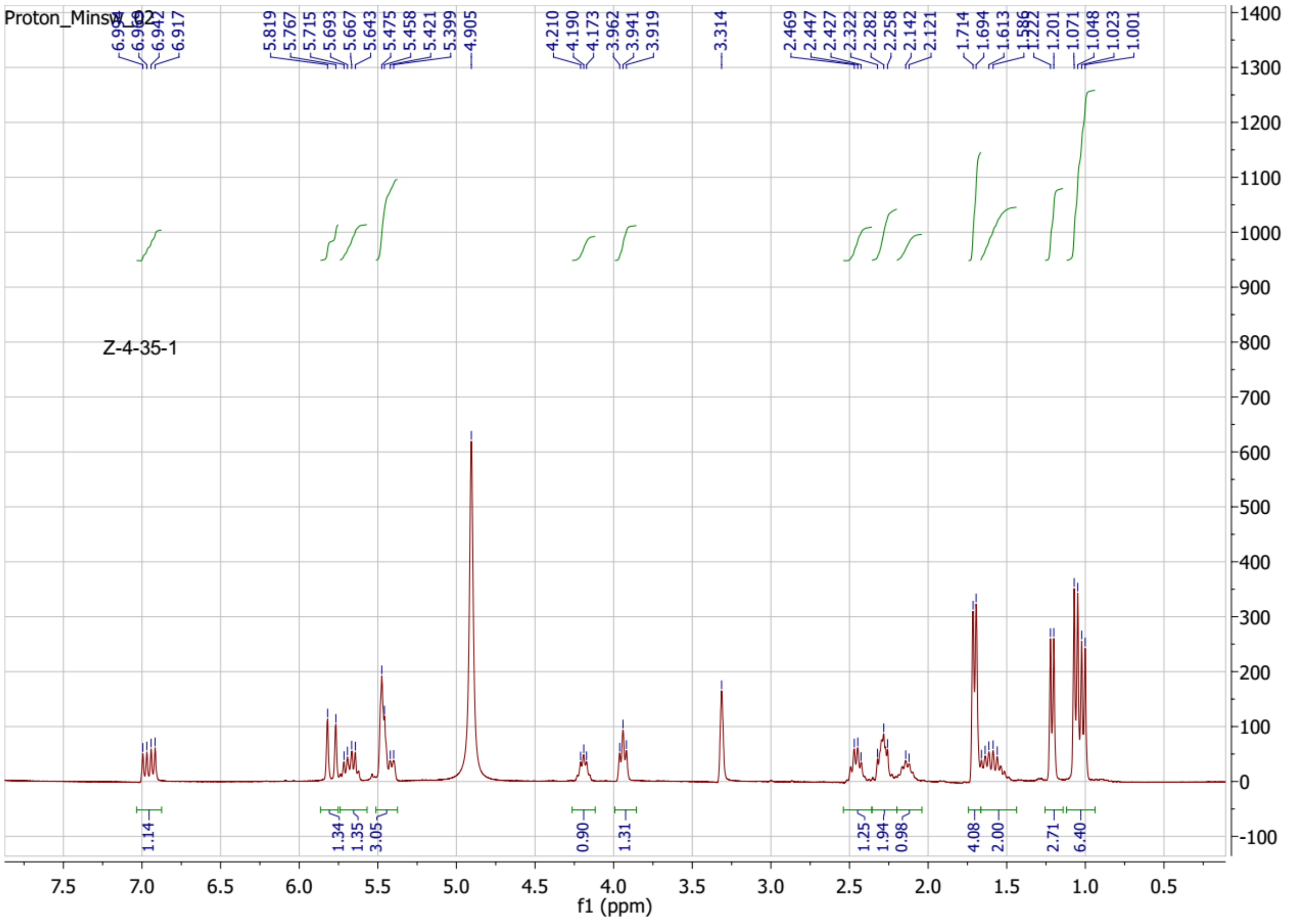

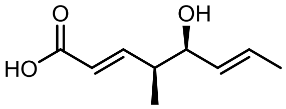

3

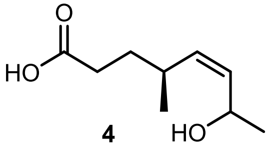

4

Figure S15. ^{1}H NMR (500 MHz, CD_3OD) of compound **3**.

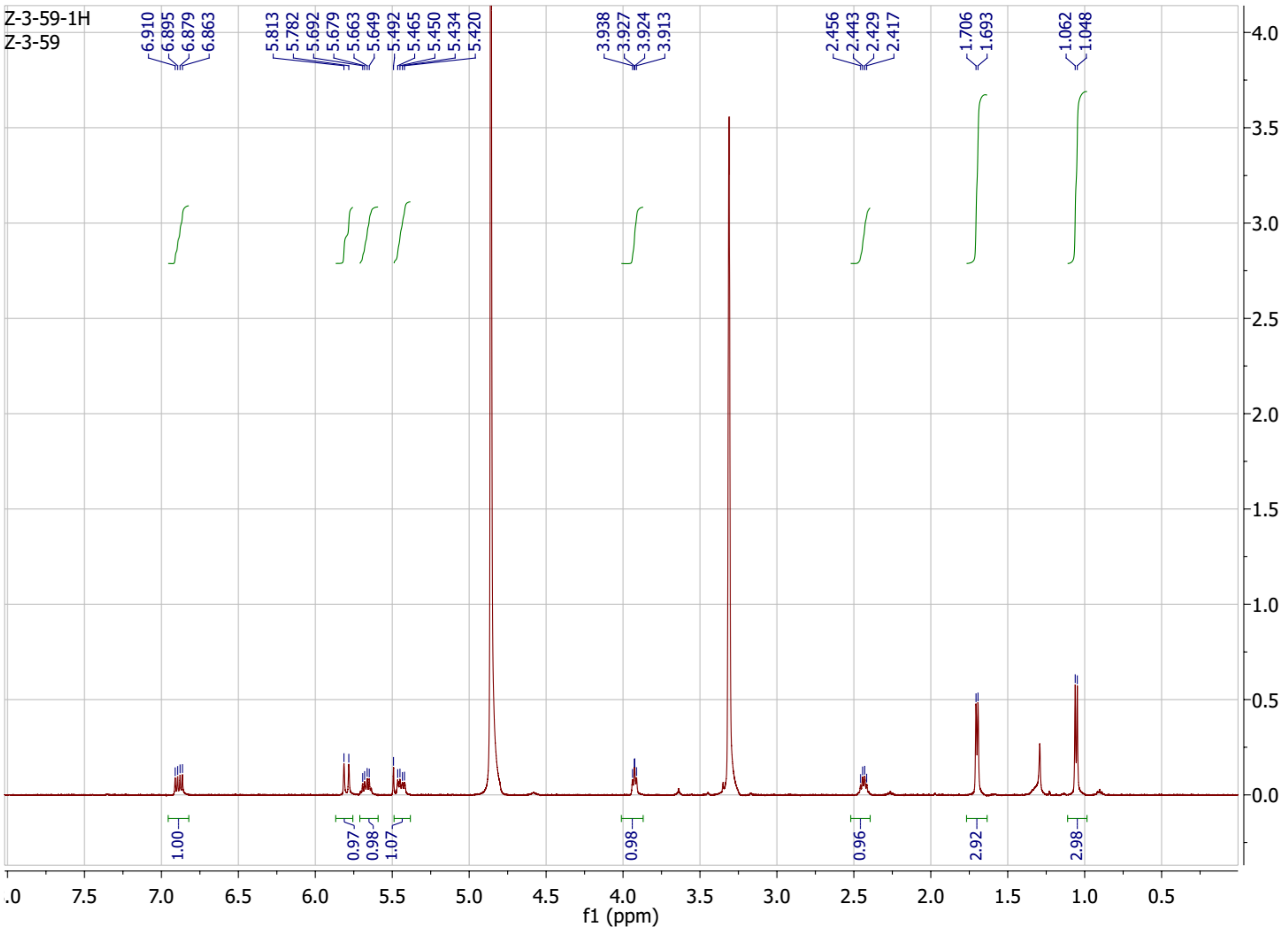

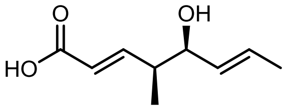

3

Figure S16. ^{13}C NMR (75 MHz, CD_3OD) of compound **3.**

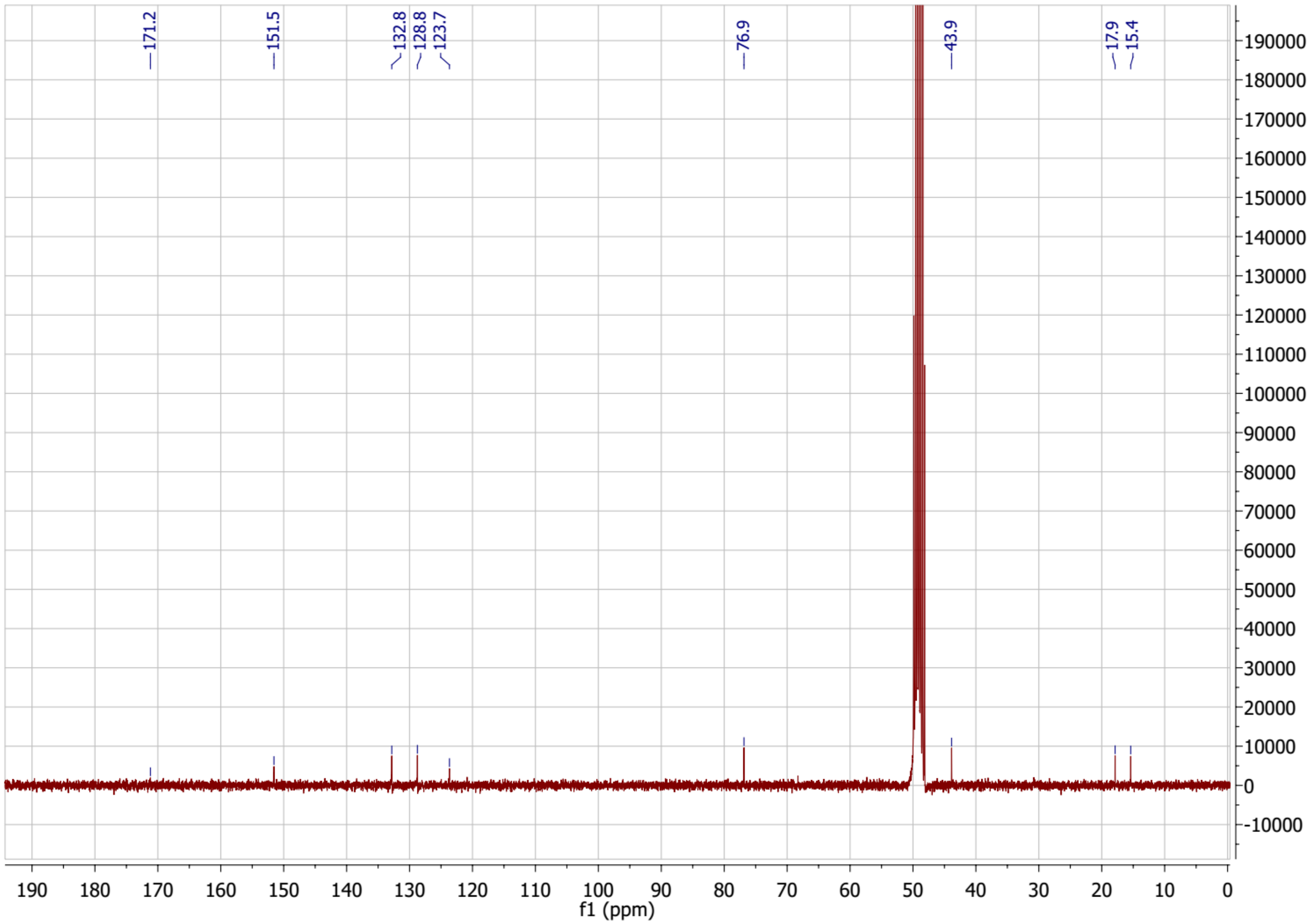

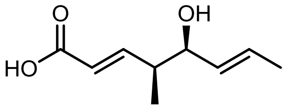

3

Figure S17. ^{1}H-^{1}H COSY (500 MHz, CD_3OD) of compound **3**

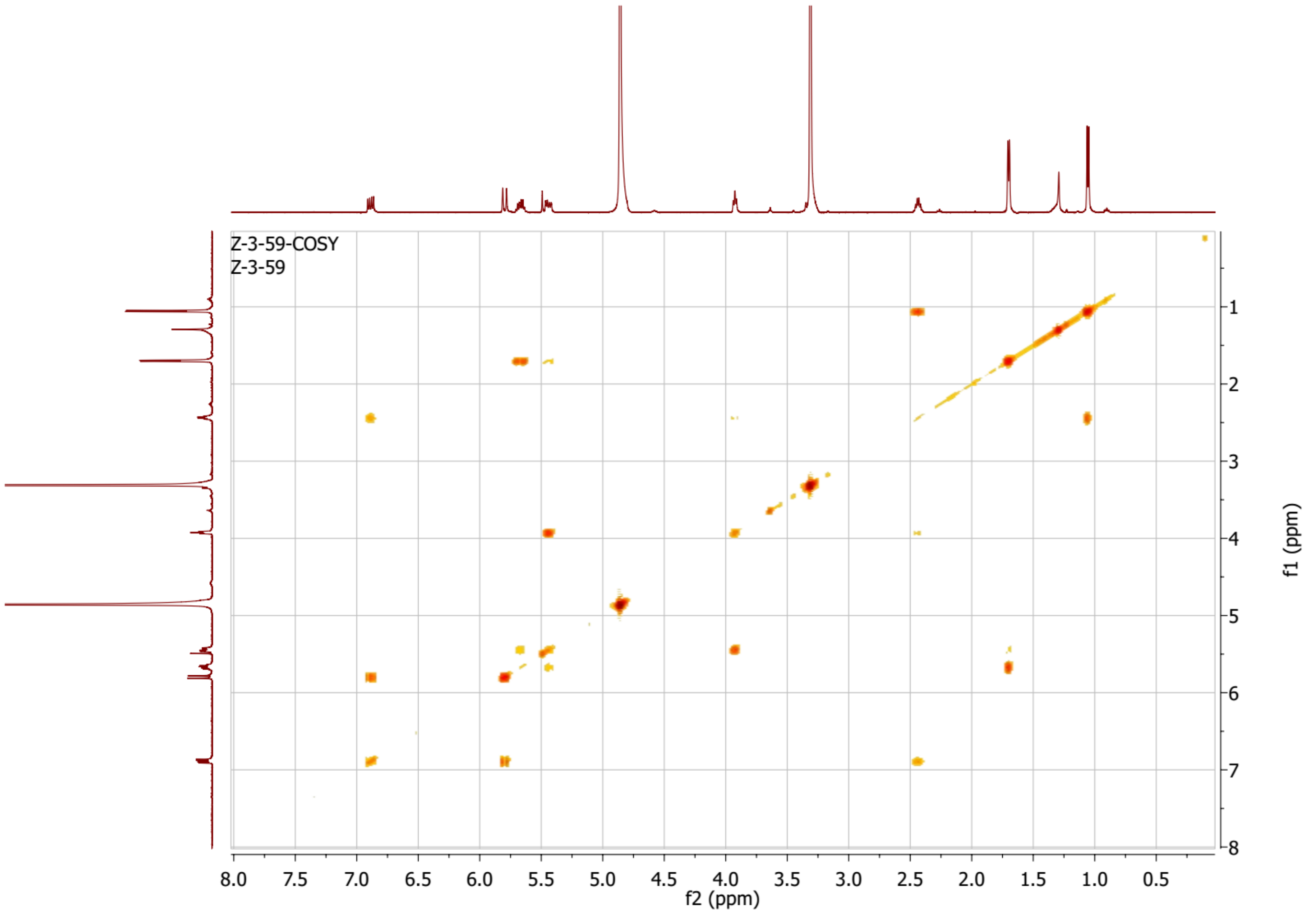

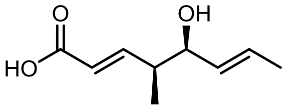

3

Figure S18. HSQC (500 MHz, CD_3OD) of compound **3**

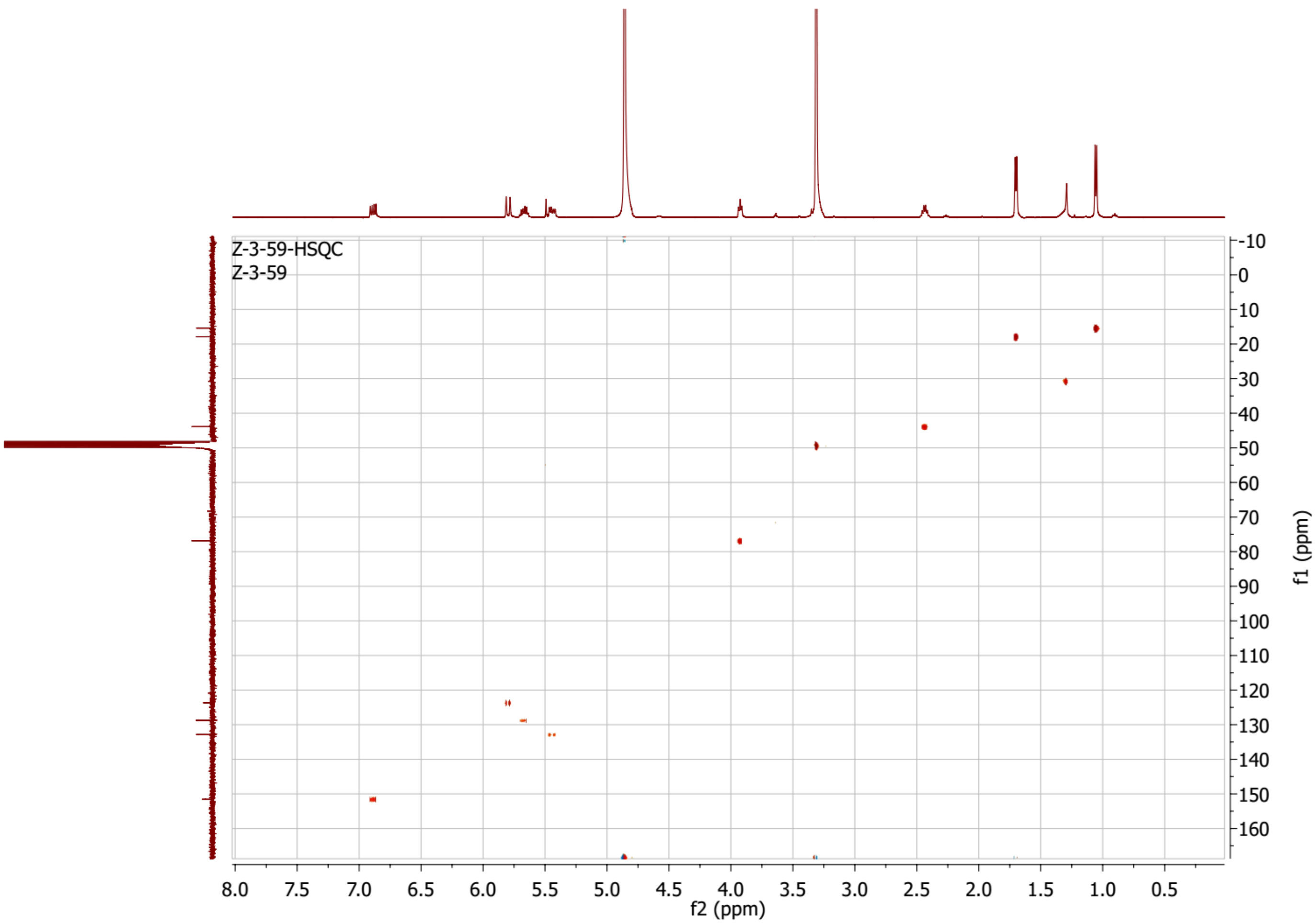

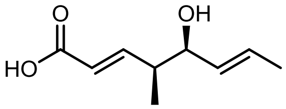

3

**Figure S19.** HMBC (500 MHz, CD_3OD) of compound **3**.	
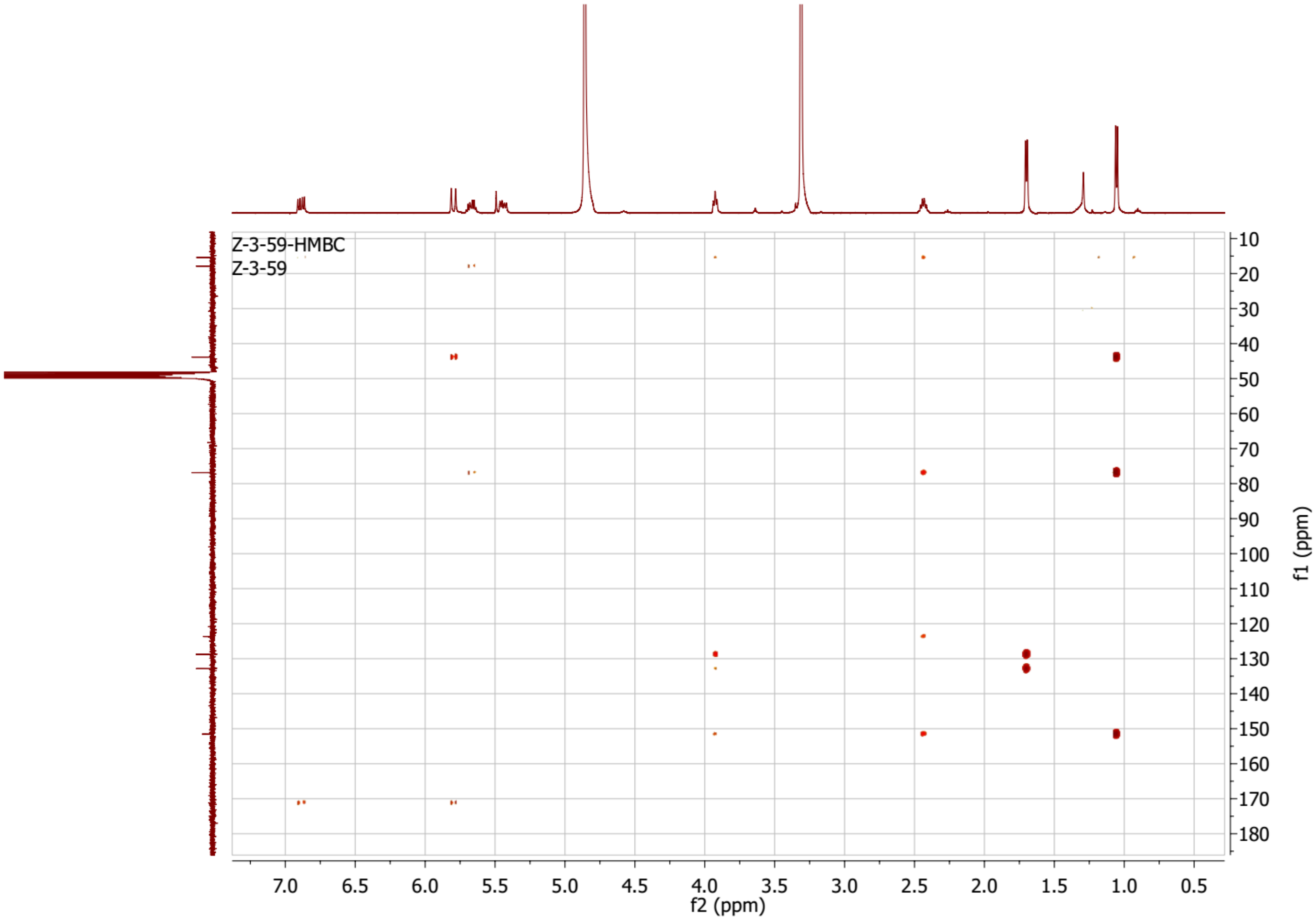	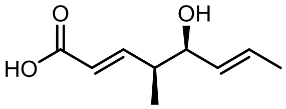 **3**

**Figure S20.** ^{1}H NMR (500 MHz, CD_3OD) of compound **4**.	
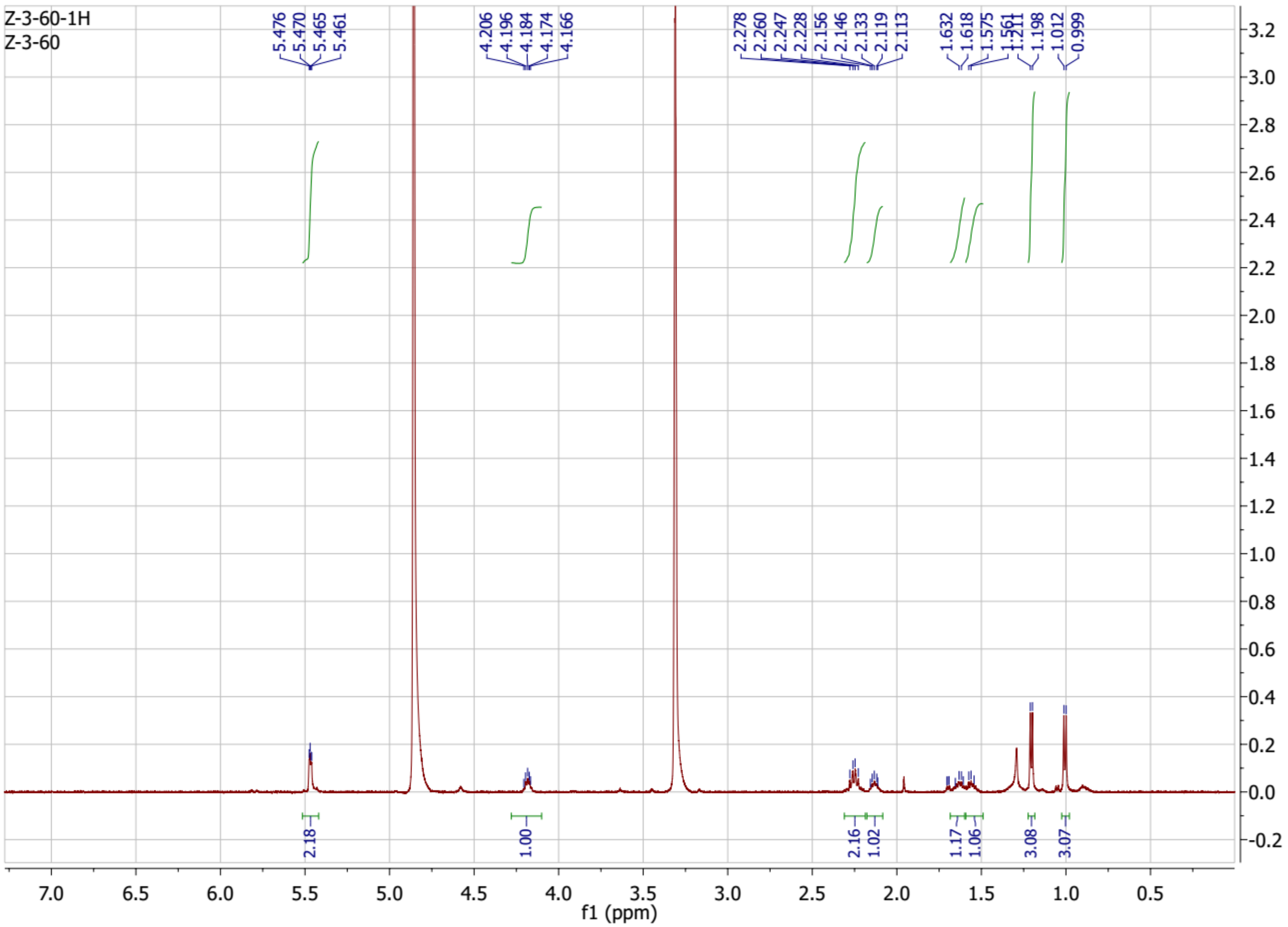	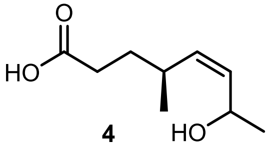 **4**

**Figure S21.** ^{13}C NMR (75 MHz, CD_3OD) of compound **4**	

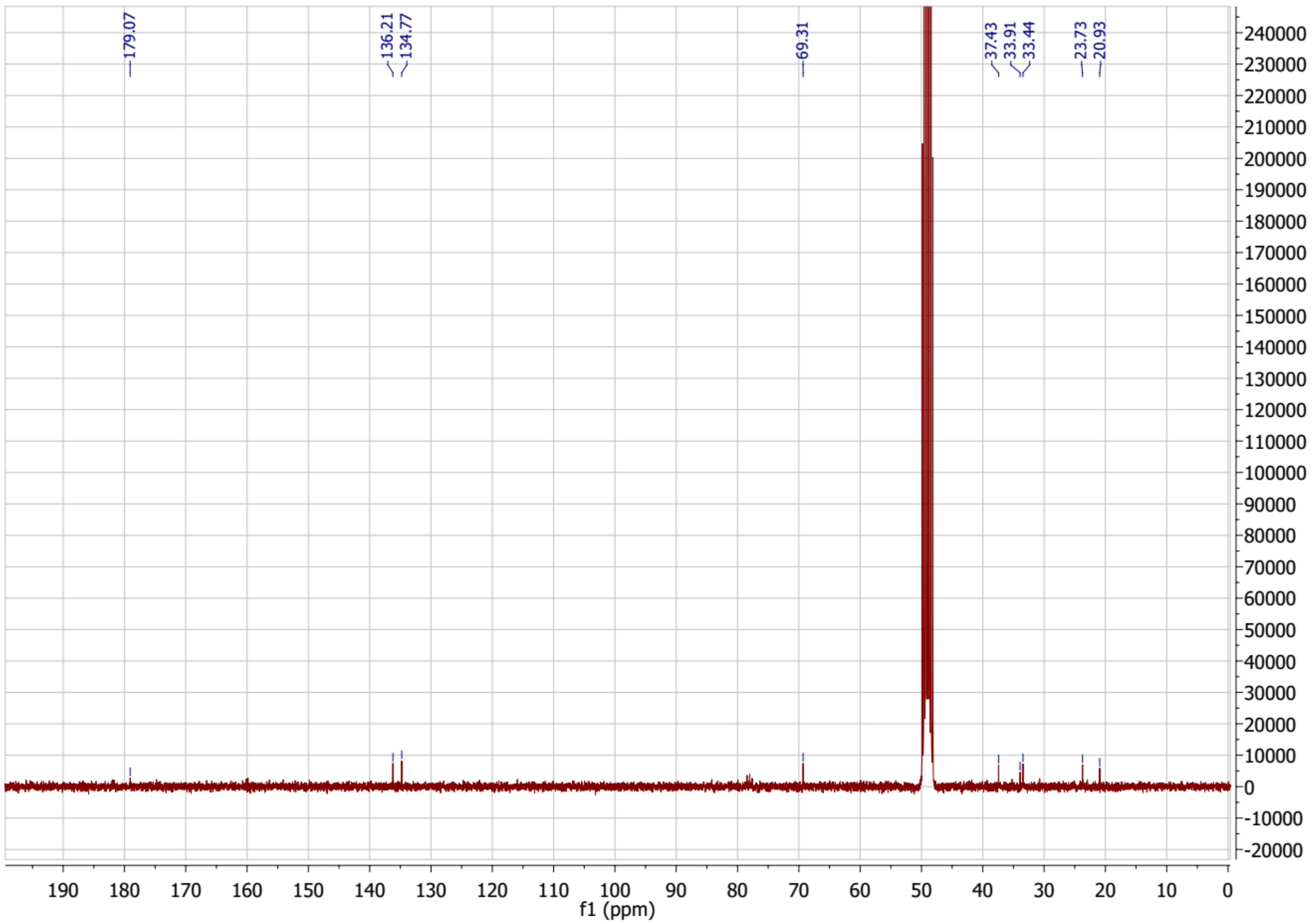

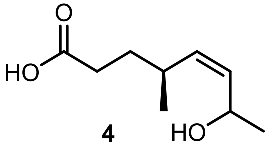

4

Figure S22. ^{1}H-^{1}H COSY (500 MHz, CD_3OD) of compound **4**	
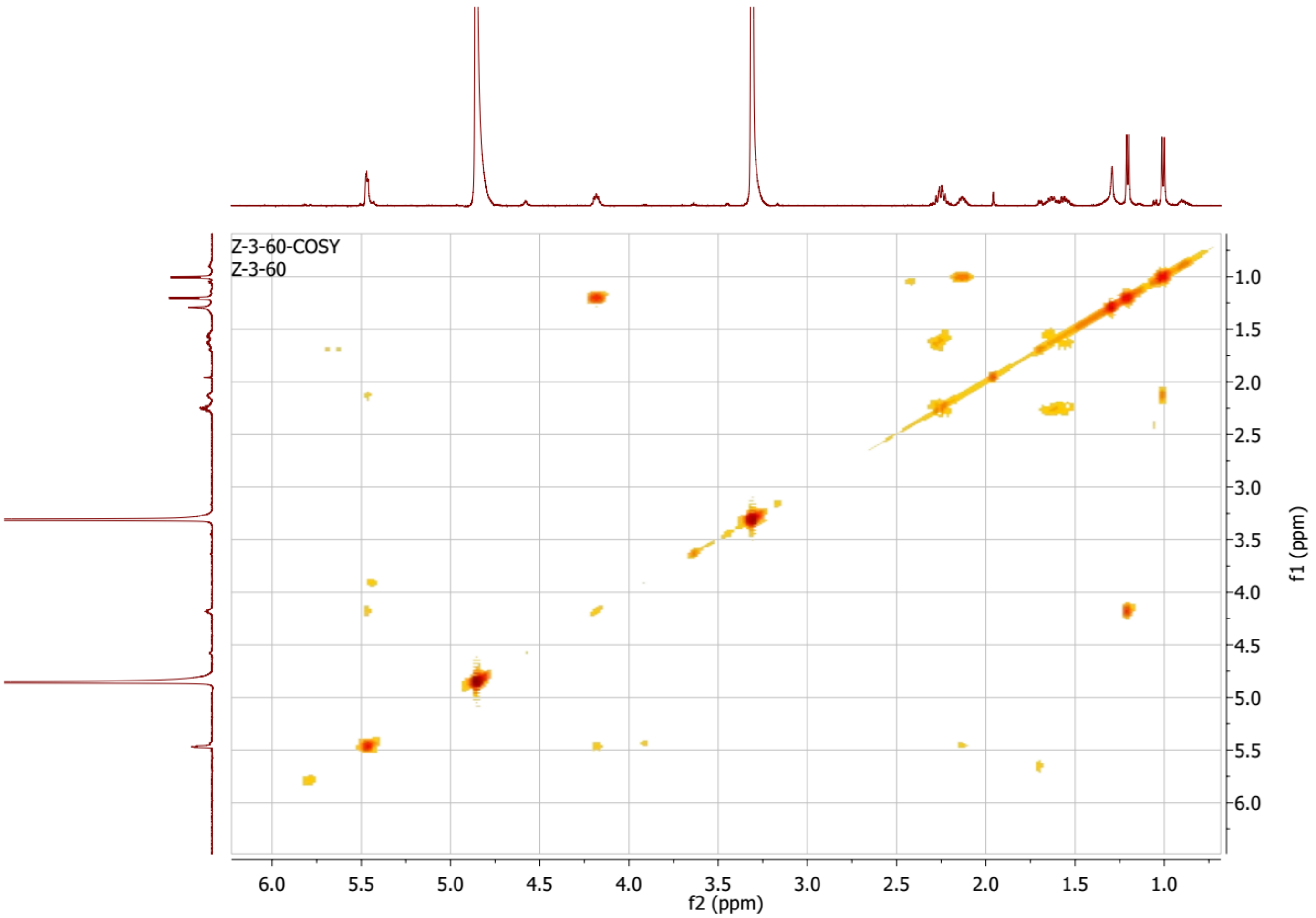	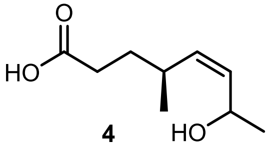 **4**

Figure S23. HSQC (500 MHz, CD_3OD) of compound **4**.

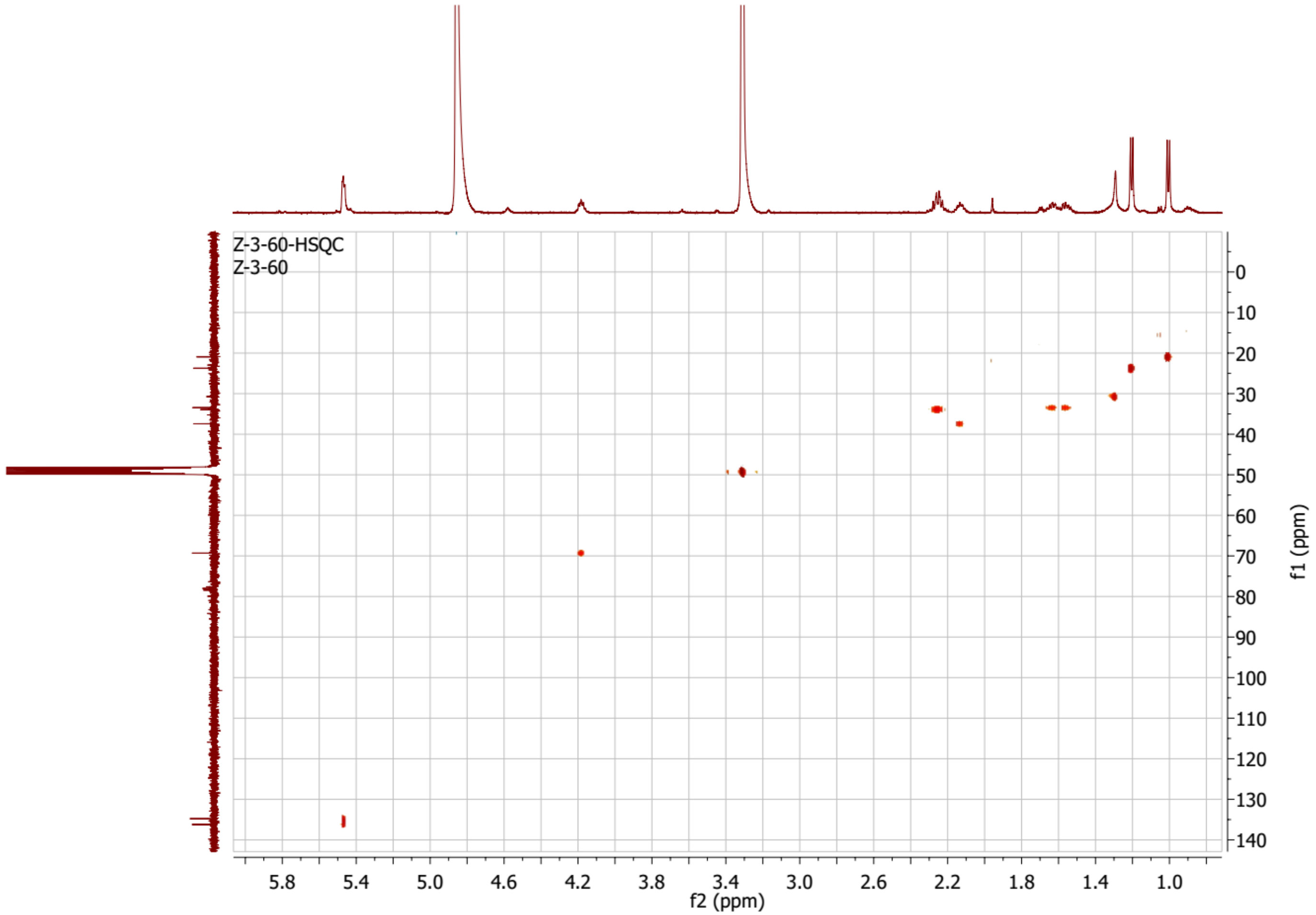

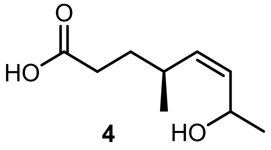

4

Figure S24. HMBC (500 MHz, CD3OD) of compound **4**.

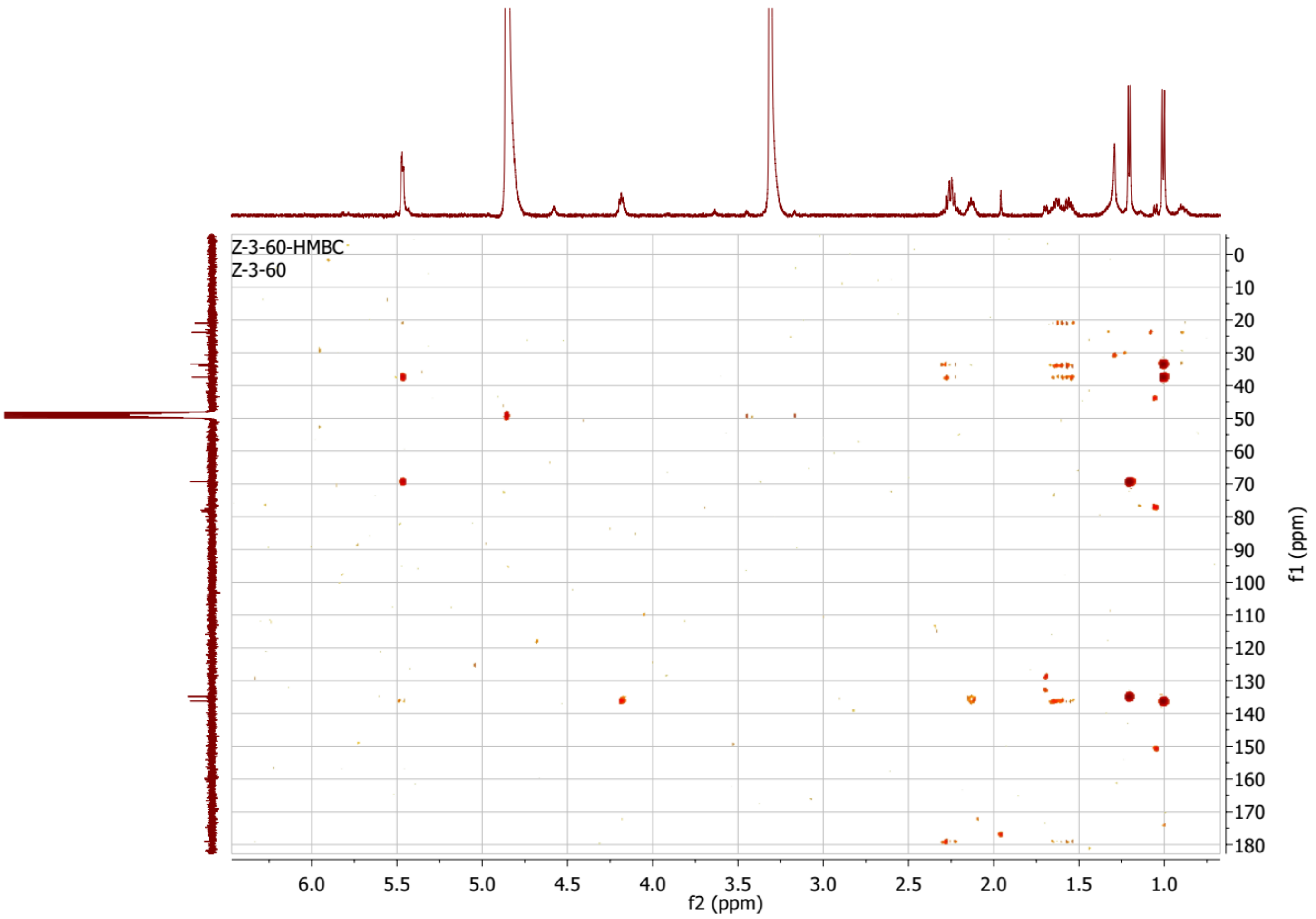

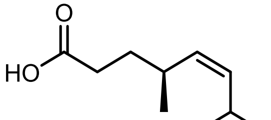

4

Figure S25. HRMS of compound **1**.

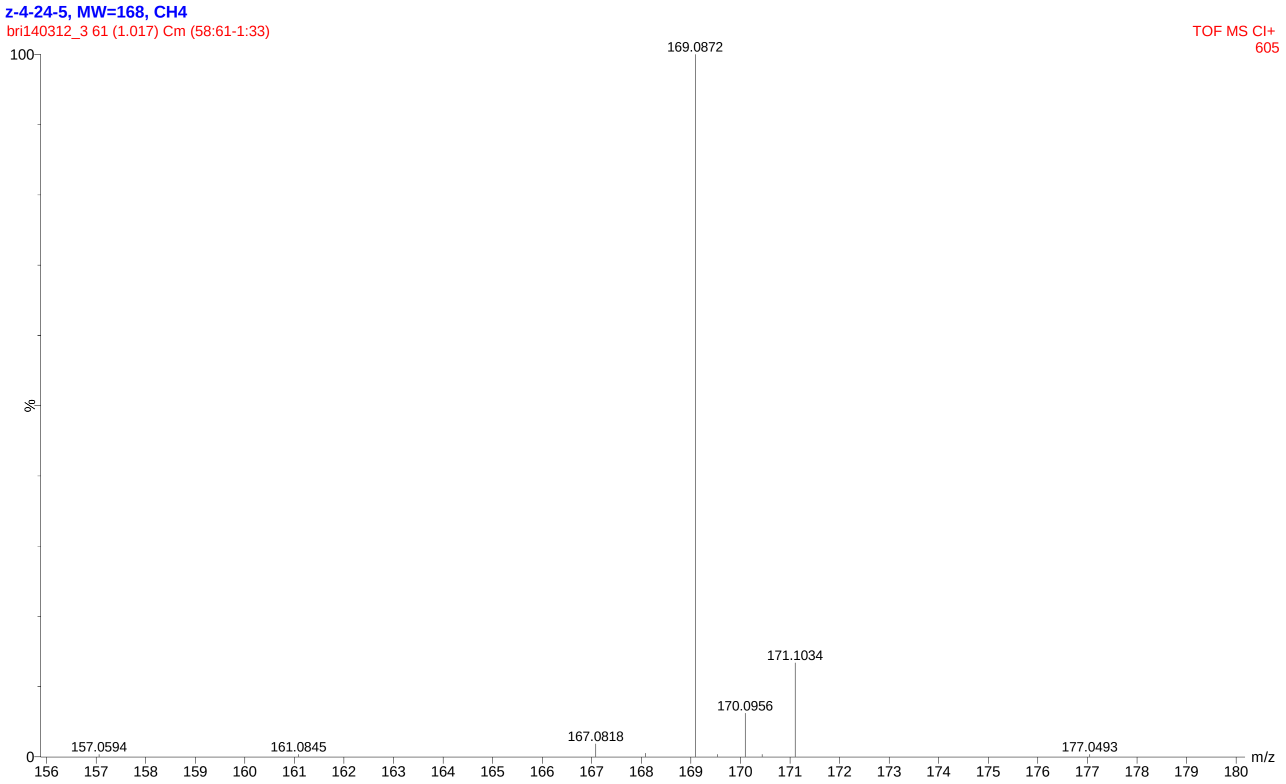

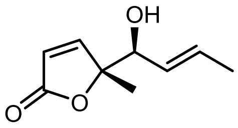

1

Figure S26. HRMS of compound **2**.

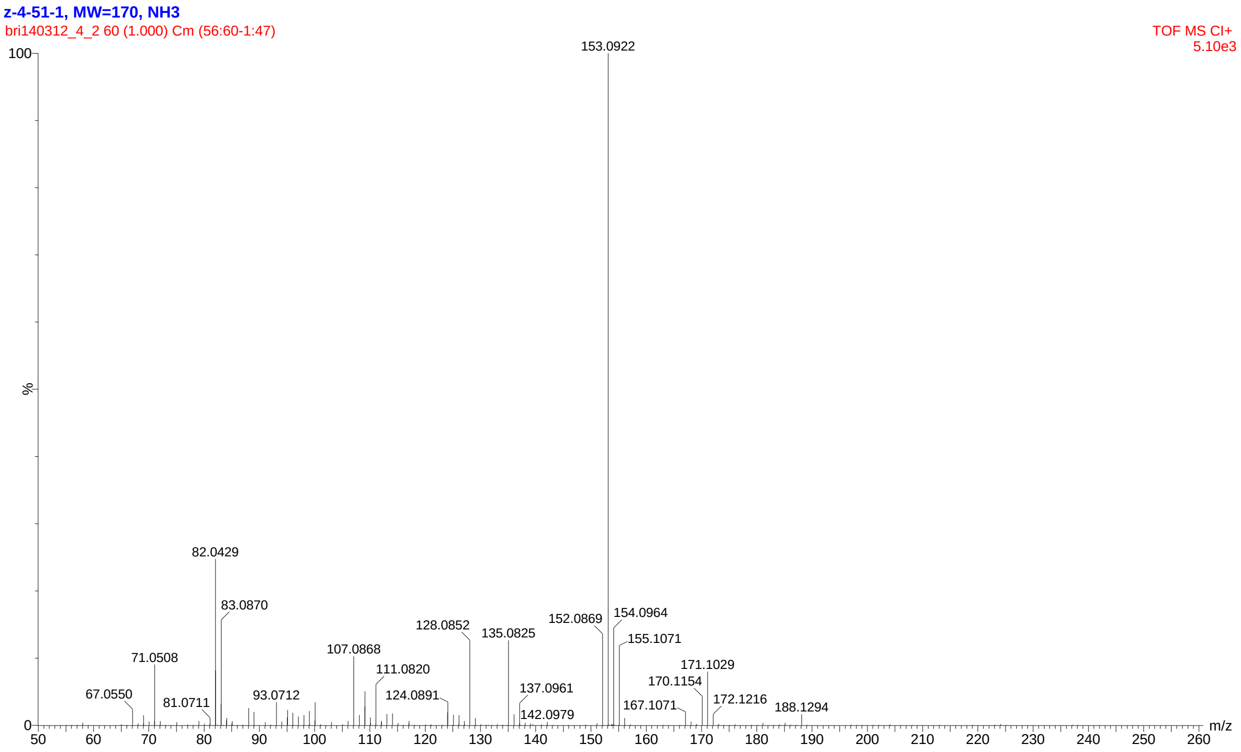

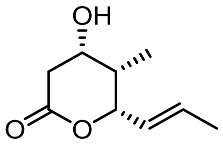

2

Figure S27. HRMS of compound **3**.

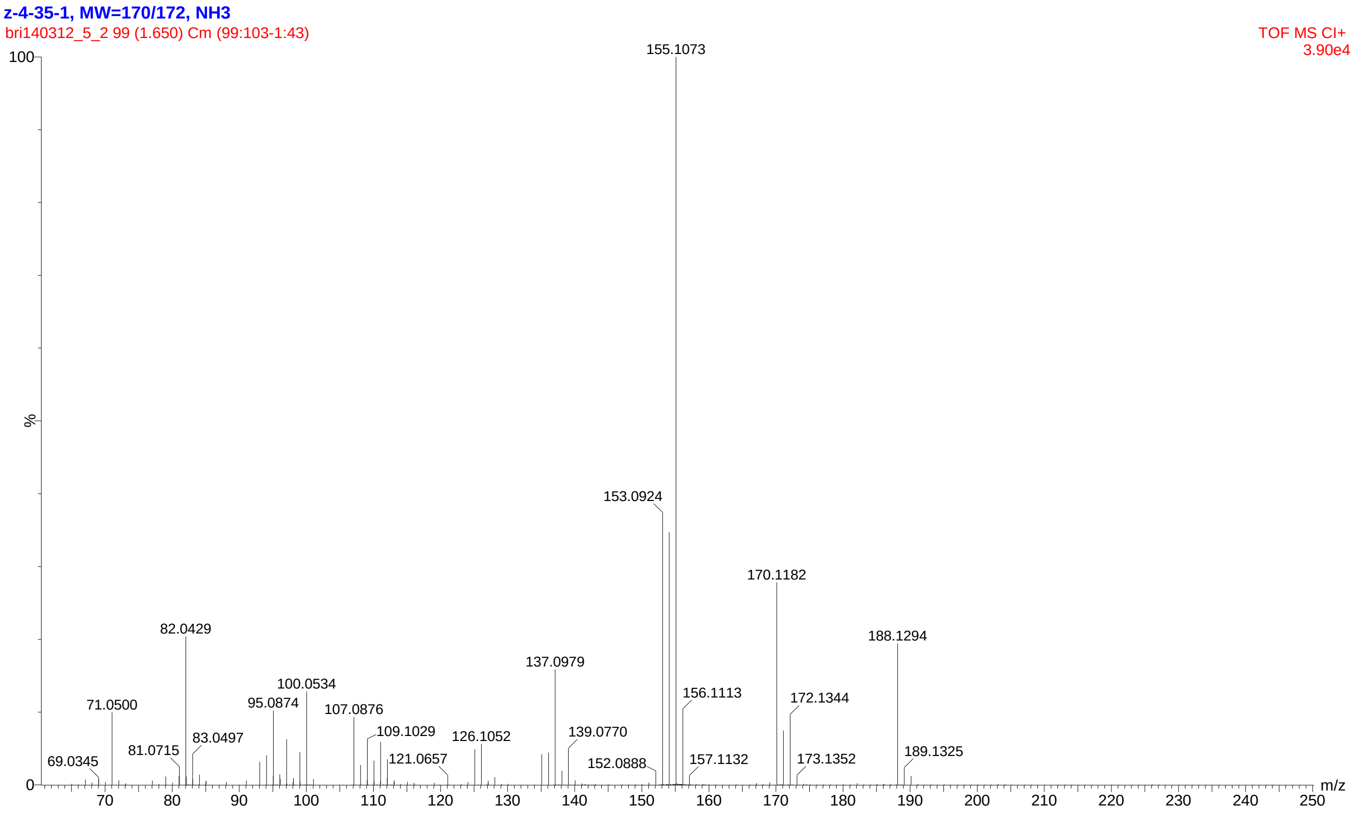

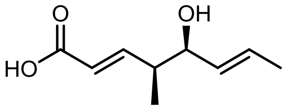

3

Figure S28. HRMS of compound **4**.

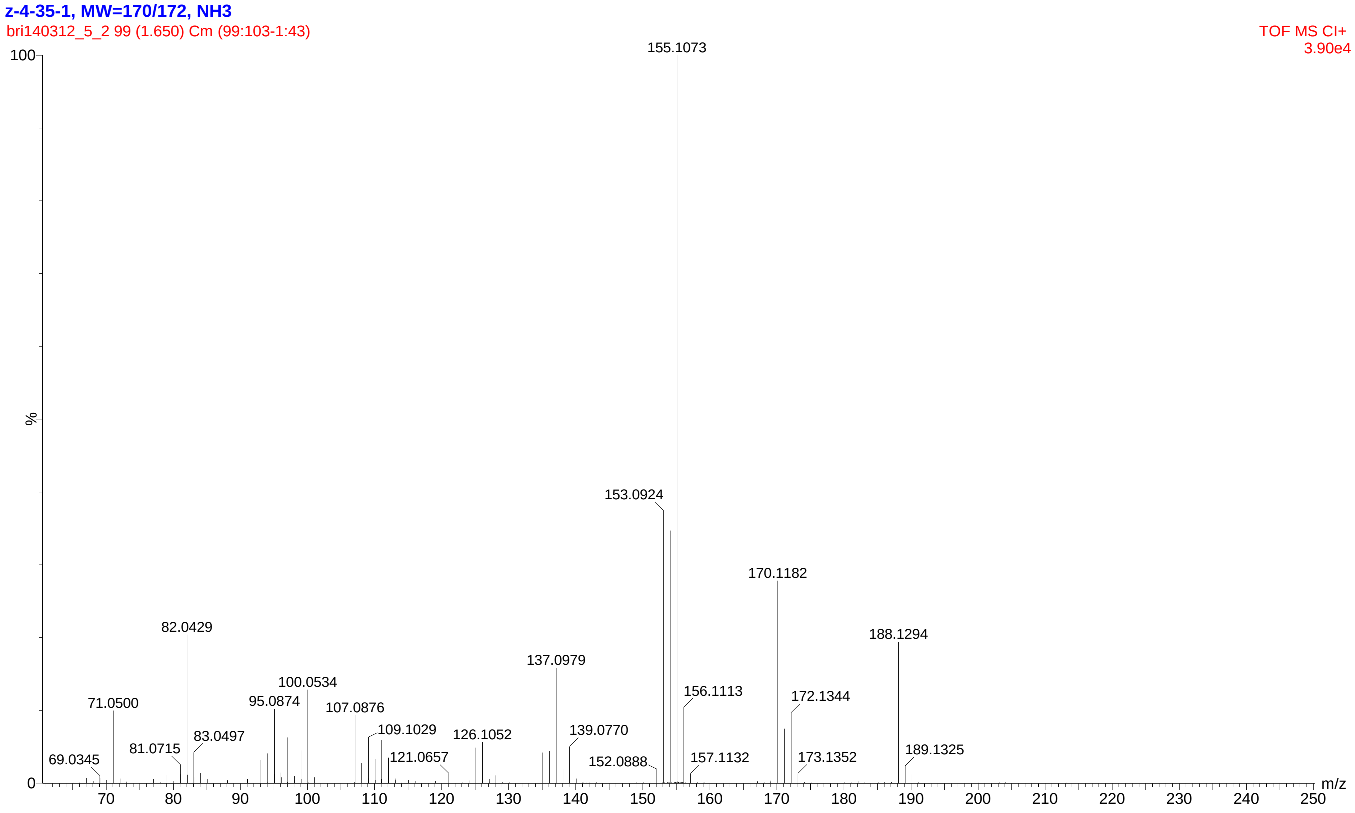

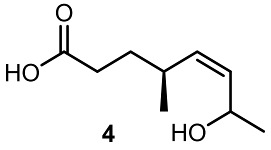

4

Figure S29. IR of compound **1**.

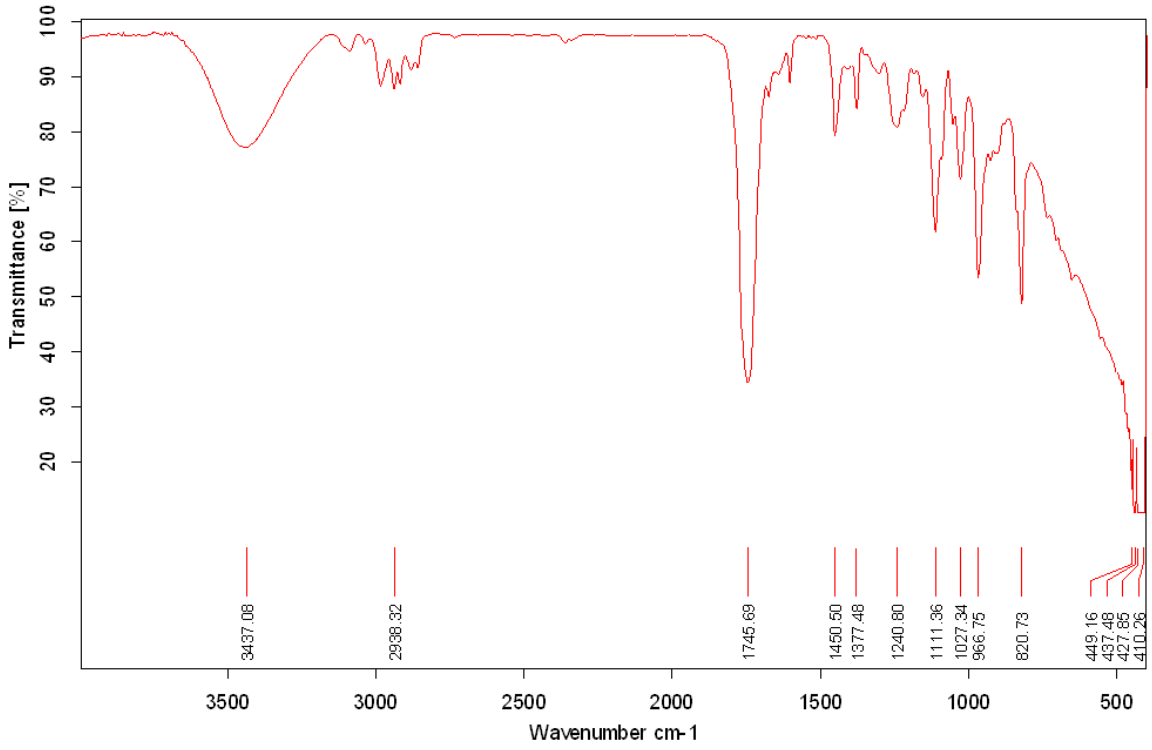

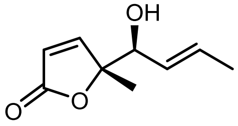

1

Figure S30. IR of compound **2**.

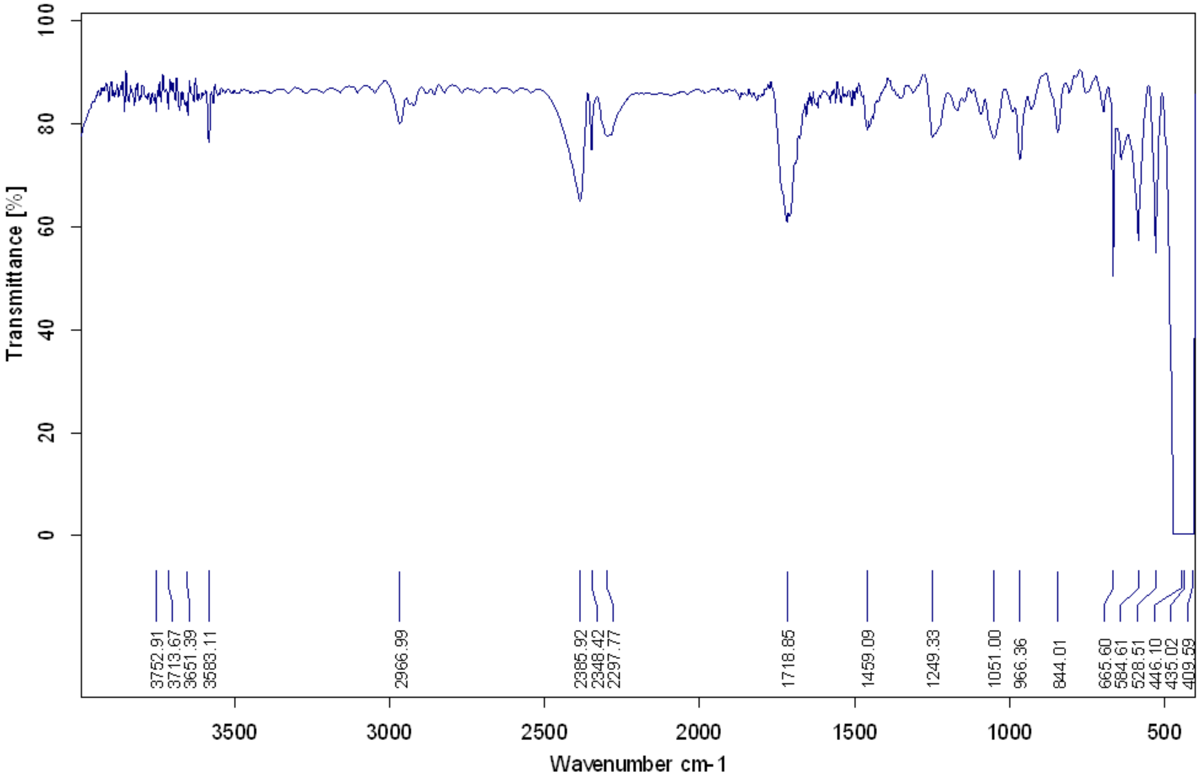

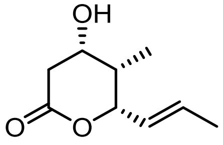

2

Figure S31. IR of compound **3**.

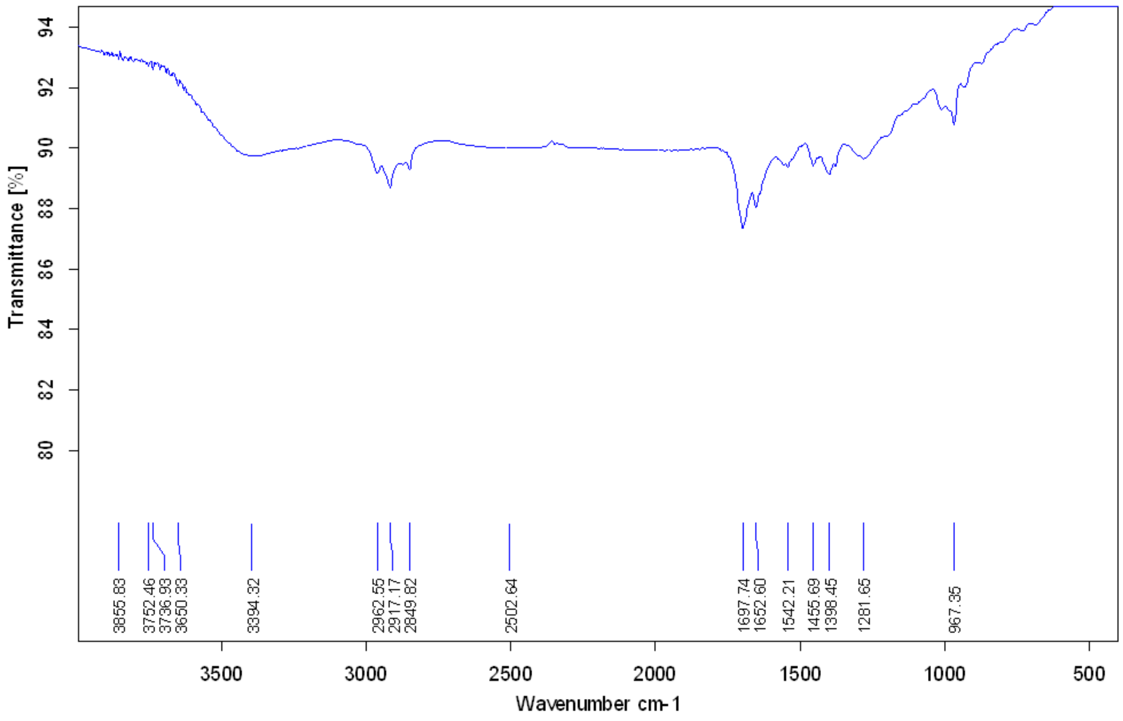

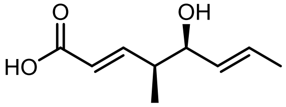

3

Figure S32. IR of compound **4**.

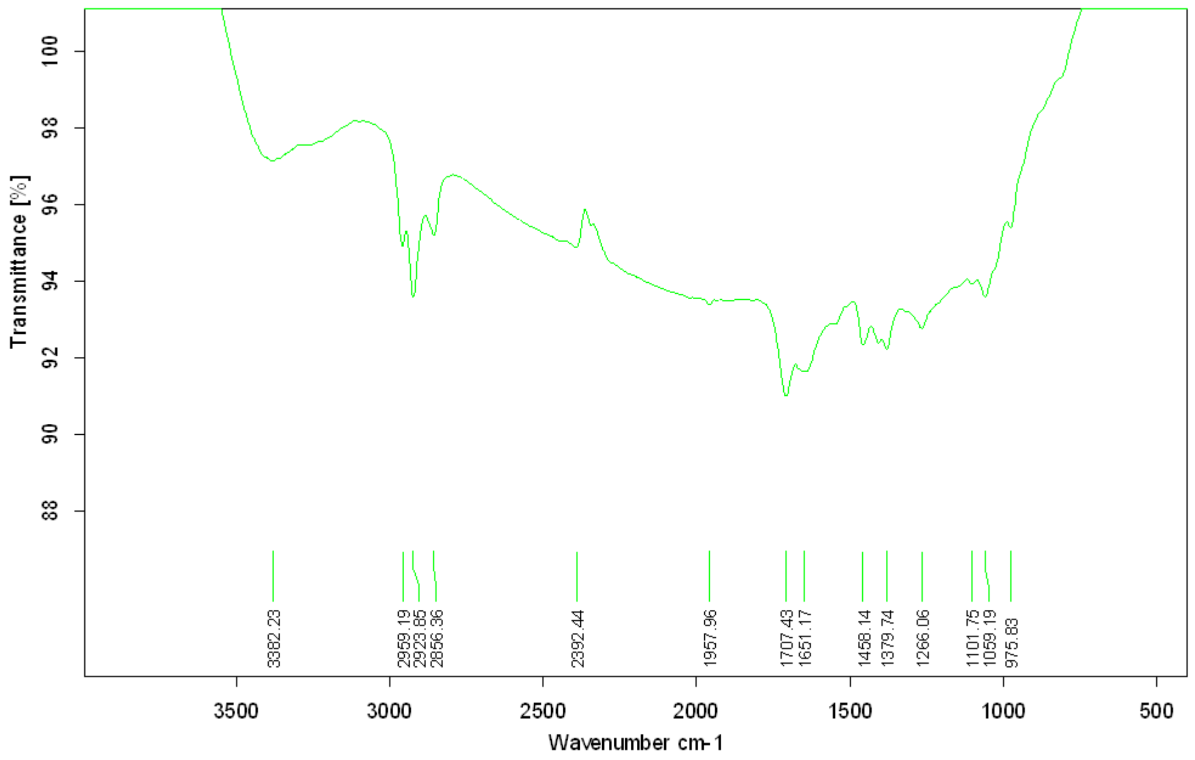

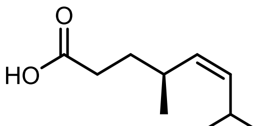

4
